# Association Analysis of SSR Markers with Phenology, Grain, and Stover-Yield Related Traits in Pearl Millet (*Pennisetum glaucum* (L.) R. Br.)

**DOI:** 10.1155/2014/562327

**Published:** 2014-01-02

**Authors:** Baskaran Kannan, Senthilvel Senapathy, Arcot Gajaraj Bhasker Raj, Subhash Chandra, Arunachalam Muthiah, Arun Prabhu Dhanapal, Charles Thomas Hash

**Affiliations:** ^1^International Crops Research Institute for the Semi-Arid Tropics (ICRISAT), Patancheru, Hyderabad, Andhra Pradesh 502324, India; ^2^Agronomy Department, University of Florida, P.O. Box 110300, Gainesville, FL 32611, USA; ^3^Directorate of Oilseeds Research, Rajendranagar, Hyderabad, Andhra Pradesh 500030, India; ^4^Department of Primary Industries, Tatura, VIC 3616, Australia; ^5^Tamil Nadu Agricultural University, Coimbatore, 641003 Tamil Nadu, India; ^6^1-31 Agriculture Building, University of Missouri, Columbia, MO 65211, USA; ^7^International Crops Research Institute for the Semi-Arid Tropics (ICRISAT), ICRISAT Sahelian Center, BP 12404, Niamey, Niger

## Abstract

Pearl millet is a staple food crop for millions of people living in the arid and semi-arid tropics. Molecular markers have been used to identify genomic regions linked to traits of interest by conventional QTL mapping and association analysis. Phenotypic recurrent selection is known to increase frequencies of favorable alleles and decrease those unfavorable for the traits under selection. This study was undertaken (i) to quantify the response to recurrent selection for phenotypic traits during breeding of the pearl millet open-pollinated cultivar “CO (Cu) 9” and its four immediate progenitor populations and (ii) to assess the ability of simple sequence repeat (SSR) marker alleles to identify genomic regions linked to grain and stover yield-related traits in these populations by association analysis. A total of 159 SSR alleles were detected across 34 selected single-copy SSR loci. SSR marker data revealed presence of subpopulations. Association analysis identified genomic regions associated with flowering time located on linkage group (LG) 6 and plant height on LG4, LG6, and LG7. Marker alleles on LG6 were associated with stover yield, and those on LG7 were associated with grain yield. Findings of this study would give an opportunity to develop marker-assisted recurrent selection (MARS) or marker-assisted population improvement (MAPI) strategies to increase the rate of gain for pearl millet populations undergoing recurrent selection.

## 1. Introduction

Pearl millet (*Pennisetum glaucum* (L.) R. Br. = *Cenchrus americanus* (L.) Morrone) is a highly cross-pollinated, diploid, photosynthetically efficient C4 monocot. It is globally the sixth most important cereal crop grown on >29 m ha annually, primarily on drought-prone and impoverished soils in the semiarid regions of Africa and the Indian subcontinent [1]. This millet provides staple food grain and a source of feed, fodder, fuel, and construction material and supports millions of poor rural families in the hottest and driest drought-prone arid and semiarid regions of Africa and the Indian subcontinent where rain-fed agriculture is practiced. Grain yields are generally low, mainly because this crop is often cultivated under low input conditions of subsistence farming systems in marginal environments. However, pearl millet is also grown under irrigation under high-temperature conditions (in India), as a mulch crop to protect soils in minimum-tillage soy production systems in the *Cerrados* of Brazil and as a temporary pasture under high temperature conditions on the Coastal Plain of the southeastern United States. Improved open-pollinated pearl millet cultivars are primarily developed by recurrent selection. Open-pollinated cultivars of this highly cross-pollinated seed-propagated crop exhibit intravarietal variability that contributes to yield stability by reducing risks of crop failure due to drought and its interaction with low soil fertility, downy mildew, striga, blast, or rust and permits reselection to improve their adaptation to particular target environments.

The International Crops Research Institute for the SemiArid Tropics (ICRISAT) at Patancheru, India, has developed large numbers and diverse ranges of maintainer lines (B-lines) and restorer lines (R-lines) of pearl millet over the past three decades [2, 3]. Genetic diversity analysis of elite pearl millet inbred lines and landraces using RFLP, RAPD, and SSR markers is well understood [2–6]. Molecular markers-based genetic maps facilitate applied genetics and breeding programs of pearl millet. However, compared to other cereals such as rice, sorghum, maize, wheat, and barley, there has been relatively little research on the development and application of molecular-markers-based genetic maps in pearl millet [7].

Integrated genetic maps involving molecular and phenotypic markers provide a direct means for investigating the number of genes influencing a trait, their location along the chromosomes, and the effects of variation in their dosage [8]. Marker-based genetic linkage map was originally based upon codominantly inherited RFLP markers has been extended and supplemented with a moderate number of mapped pearl millet SSR markers [9–11]. Recent reports of additional SSR, SSCP-SNP, and DArT markers for pearl millet have allowed further saturation and extension of this genetic linkage map [12–16]. As SSR markers are PCR-compatible and codominantly inherited, and can be multiplexed for simultaneous marker genotype characterization at a number of loci within or across linkage groups, their development has greatly facilitated linkage map construction and use in plant breeding.

The rate of genetic enhancement of quantitative traits like grain and stover yields (and their components) is slow because of their complex inheritance, involvement of many physiological processes, and strong influence of genotype × environment (G × E) interactions [17]. Appropriate integration of molecular technology into crop breeding schemes provides excellent opportunities to deal with challenges. The majority of current work in this area is directed towards identifying genomic regions of interest to facilitate marker-assisted selection. Grain yield varies continuously within a segregating population, even in improved open-pollinated populations in which selection has increased the frequency of favorable alleles at many loci. While conventional mapping-population-based QTL mapping has proved useful in identifying loci for many important specific qualitative and quantitative traits in plant species, it is constrained by its low resolution typically in the range of 10–30 cM [18] and the limited number of segregating alleles at any locus.

Association mapping, based on linkage disequilibrium (LD) is expected to achieve a higher resolution as it exploits historical recombinations and targets multiple alleles at individual loci to detect marker-phenotype associations [19, 20] to identify genomic regions linked to a wider range of phenotypic traits. Very few association studies have been carried out in pearl millet, largely because of the typically high level of heterozygosity and heterogeneity within landrace germplasm accessions and improved open-pollinated varieties. Molecular and phenotypic diversity in pearl millet germplasm from west and central Africa and their relation to geographical and environmental parameters by population structure have been studied [21]. Significant associations between light perception gene *PHYC* and flowering time, spike length, and stem diameter among a panel of inbred lines were detected using SSR and AFLP markers [22]. Recently, polymorphism at the MADS-box gene *PgMADS11* associated with flowering time variation was reported [23]. Therefore, this exploratory study was aimed at assessing the ability of modest numbers of codominant markers to identify genomic regions responding to selection in a conventional pearl millet population improvement program and to identify genomic regions associated with phenology, grain, and stover yield-related traits.

## 2. Materials and Method

### 2.1. Genetic Materials for Full-Sib Progenies Development

Pearl millet composite “CO(Cu) 9” and four of its immediate progenitor populations (ICMP 87750, ICMP 91751, ICMV 93752, and UCC 23) were used in this study. ICMP 87750 (SRC II C_0_) is a random-mated half-sib composite bulk generated by crossing 62 S_2_ selections of the ICRISAT smut resistant composite (SRC) C_3_-cycle with 71 S_2_ selections of the ICRISAT intervarietal composite (IVC) C_7_-cycle. ICMP 91751 is a random-mated full-sib (FS) bulk composite derived from 51 S_1_ progenies of ICMP 87750 selected from 1197 such S_1_ progenies by culling weaker progenies and selecting for medium maturity, shorter plant height, and long, compact panicles based on their performance at ICRISAT-Patancheru (17°N). ICMV 93752 was derived from ICMP 91751 by imposing progeny-based selection pressure for uniform earlier maturity, longer panicles, and higher grain yield with selection primarily based on June sowings. UCC 23 was derived from ICMV 93752 by mass selection for higher yield and agronomic score. UCC 23 was fine-tuned by a further cycle of mass selection to synthesize improved composite “CO(Cu) 9.” These last two cycles of recurrent selection were carried out at TNAU-Coimbatore (11°N) with selection primarily based on October sowings.

The seeds of each population bulk were sown in 40-row plots of 4 m length at ICRISAT-Patancheru during the 2005 summer season (January—April). Row-to-row and plant-to-plant spacing were maintained at 60 cm and 20 cm, respectively. FS progenies of each population were synthesized by plant × plant crosses. Each crossed panicle was harvested and threshed separately. Circa 200 FS progenies were developed from each population and a subset of 50 from each population was selected randomly for field evaluation and marker genotyping.

### 2.2. Phenotypic Analysis

The FS progenies were evaluated at ICRISAT-Patancheru during the rainy (June—September) and the summer (February—May) seasons in 2005 and 2006, respectively. A total of 260 entries, in which 50 FS progenies from each of the five populations and 10 check entries from composite “CO(Cu) 9”, were included in this trial. Both seasons experiments were laid out in 13 × 20 alpha-lattice designs with three replications. Inter- and intrarow spacing was maintained at 75 cm and 20 cm, respectively. Initially over-sown plots were thinned within 2 weeks of seedling-emergence to a uniform stand of approximately 8 plants m^−2^. Flowering time (FT) was recorded as days to 75% stigma emergence on main stems in a plot. Plant height (PH), panicle length (PL), and panicle diameter (PD) were measured from main stems of five representative plants of each entry in a plot. At harvest, data were recorded from the harvested area on plant numbers (PC), head numbers (HC), fresh stover yield (FSY), and effective tiller number (ET) calculated as the ratio HC/PC. Panicle yield (PY), grain yield (GY), and 1000-grain mass (TGM) were recorded after oven drying for approximately 24 h; stover dry matter yield (SDMY) was estimated from plot FSY using the fresh and dry weights of a chopped subsample of stover from each plot. Biomass yield (BMY) was calculated as PY + SDMY on a plot basis and panicle threshing percentage (PTP) as (GY/PY) ×100. Panicle grain number (PGN) was derived from these primary data as (GY/HC)/(TGM/1000). Vegetative growth index (VGI) was calculated as the ratio BMY/(FT + 10). Harvest index (HI) was estimated as the ratio (GY/BMY) × 100. For traits measured on individual plants, the phenotypic data were analyzed as means of the five individual plants from each plot. The traits measured on a complete plot basis were converted to a square meter basis prior to analyses.

### 2.3. Genotypic Analysis

Thirty-four selected SSR primer pairs detecting single codominant loci distributed across all seven pearl millet linkage groups were used (Supplementary file 1 see in Supplementary Material available online at http://dx.doi.org/10.1155/2014/562327). PCR conditions for circa 70 pearl millet SSR primer pairs were optimized for the concentrations of primers, template DNA, Mg^++^, dNTP, enzyme, and annealing temperature. Out of 70 SSR primer pairs tested with DNA mixtures from two pairs of genetically diverse pearl millet inbreds, 34 were found to produce expected amplification profiles (indicating minimal allelic competition during the PCR reaction) and were chosen for genotyping FS progeny sets from all five populations. A cross between two non-identical heterozygous diploids would produce an FS progeny segregating for up to four alleles per locus and the number of segregating alleles can vary from one locus to the other. Based on their parental genetic constitutions, the five different allelic ratios that can be expected for an individual FS progeny at a single codominant SSR locus are listed in Supplementary file 2.

DNA of each FS progeny was extracted from pooled leaf tissues of at least 20 seedlings using a modified CTAB/*β*-mercaptoethanol method [24]. Final DNA concentrations were normalized to 5 ng/*μ*L using a Spectraflour Plus (Tecan, Switzerland) plate reader for working samples used in PCR reactions. PCR reactions were conducted in volumes of 5 *μ*L, using a GeneAmp PCR system 9700 (Applied Biosystems, CA, USA) thermocycler. The PCR reaction mixture contained 1.55 *μ*L of sterile distilled water, 1 *μ*L of template DNA (5 ng/*μ*L), 0.5 *μ*L of PCR buffer (10X), 0.5 *μ*L of forward and reverse primers (2 pmol), 1 *μ*L of MgCl_2_ (10 mM), 0.25 *μ*L dNTPs (2 mM), and 0.2 *μ*L Ampli Gold *Taq* DNA polymerase (0.5 U/*μ*L). A touchdown PCR program was used to amplify the DNA fragments. Initial denaturation was for 15 minutes at 94°C. This was followed by 10 cycles of denaturation for 15 seconds at 94°C, annealing at 61°C for 20 seconds (the annealing temperature for each cycle being reduced by 1°C), and extension at 72°C for 30 seconds. Subsequently, 32 cycles of denaturation for 10 seconds at 94°C, annealing at 54°C for 20 seconds, and extension at 72°C for 30 seconds were followed by a final 20-minute extension at 72°C. The PCR products were size-separated by capillary electrophoresis using an ABI Prism 3700 DNA analyzer (Applied Biosystems, CA, USA). Further fragment analysis was carried out using Genescan 3.1 and Genotyper 3.7 (Life Technologies, CA, USA) software according to manufacturer's instructions. The heights of the chromatogram peaks (representing the alleles) obtained through capillary electrophoresis were directly proportionate to the signal strengths, which in turn are determined by the amount of amplified products in the sample. Based on the heights of the chromatogram peaks, the allele frequencies were scored for PCR products of various SSR primer pairs amplified using template DNA of each FS progeny set.

### 2.4. ANOVA Analysis

The plot-level data on each trait in each season were analyzed using the following linear mixed model for an alpha-lattice design:

(1)      Yijk=μ+Ri+Bij+Gk+eijkii=1,…,3,  j=1,…,20,  k=1,…,260,

where *Y*_*ijk*_ is the plot observation corresponding to genotype *k* in incomplete block *j* of replicate *i*, *μ* is the population mean, *R*_*i*_ is the effect of replicate *i*, *B*_*ij*_ is the effect of block *j* in replicate *i*, *G*_*k*_ is the effect of genotype *k*, and *e*_*ijk*_ is the residual. The last three terms in model (1) were treated as random, each with a mean zero and its own constant variance. Residual maximum likelihood (ReML) was used to obtain estimates of variance components for the last three terms and the best linear unbiased predictions (BLUPs) for the genotypes reflecting their average phenotypic expression. This analysis, based on 260 genotypes, gave an idea of how much the 250 FS differed from the 10 control entries. Model (1) was rerun using data only from 250 FS to get an estimate of the genetic variance among these 250 FS. Model (1) was also run separately on data from each population to obtain population-specific estimates of genetic variance. Operational heritability was estimated on plot basis as *h* ^2^ = *σ*_*g*_^2^/(*σ*_*g*_^2^ + *σ*_*e*_^2^) where *σ*_*g*_^2^ is genetic variance and *σ*_*e*_^2^ is residual variance. Across season analyses were conducted by extending model (1) to include in it the effect of season (S) and the effect of genotype × season interaction (G × S). The operational heritability in this case was estimated as *h* ^2^ = *σ*_*g*_^2^/(*σ*_*g*_^2^ + *σ*_*gs*_^2^ + *σ*_*e*_^2^), where *σ*_*gs*_^2^ is G × S interaction variance. All analyses were conducted using statistical software GenStat 9 [25]. All traits reasonably satisfied the ReML assumptions of normality and constant variance as evident in GenStat's ReML diagnostic plots.

The allele frequencies were calculated as the sum of copies of a particular allele at a locus divided by the total number of individuals in the sample [26]. The informativeness of a marker was quantified through the polymorphism information content (PIC) estimated as [27]

(2) $$\begin{array}{c} \text{PIC}=1-\Sigma p_{ij}^{2}, \end{array}$$

where *p*_*ij*_ is the frequency of *j*th microsatellite allele for the marker *i*.

### 2.5. AMOVA and Population Structure

AMOVA was calculated by GeneAlEx 6.41 [28] with 1000 permutations. The population structure was inferred using the Bayesian model-based software program STRUCTURE 2.2 [29]. The length of burn-in period and the number of Markov Chain Monte Carlo (MCMC) replications after burn-in were all assigned at 100,000 with an admixture and allele frequencies correlated model. Five independent iterations of running were performed with the hypothetic number of subpopulations (*k*) ranging from 1 to 10. The correct estimation of *k* was provided by joining the log probability of data [LnP(*D*)] from the STRUCTURE output and an ad hoc statistic Δ*k* [30], which was based on the rate of change in the log probability of data between successive *k* values. Based on the correct (*k* = 4), each FS progeny was assigned to a subpopulation for which its membership value (*Q* value) was > 0.5 [29, 31], and the population structure matrix (*Q*) was generated for further analyses. The kinship matrix (*K*) of the genetic relatedness among pair-wise genotypes, with the negative value of kinship set as zero, was used.

### 2.6. Association Analysis

To account for the population structure and genetic relatedness, two statistical models were tested: (i) general linear model (GLM) without considering *Q*-matrix and *K*-matrix and (ii) MLM model with *Q*-matrix and *K*-matrix (MLM *Q* + *K*) used to correct for population structure [32–35]. Genome-wide association analyses based on these models were conducted with the software TASSEL 3.0 [36, 37]. Markers were defined as being significantly associated with traits on the basis of their significant association threshold (−Log *P* ≥ 2.00; *P* ≤ 0.01) for GLM and MLM as evidenced from previous works [33, 38].

## 3. Results

### 3.1. Analysis of Variance and Mean Phenotypic Performance

The ReML analyses results for grain and stover yield-component traits are presented in Table 1. Estimates of genetic variance among FS progenies were significant for all traits, in individual season as well as across seasons. For flowering time, plant height, stover dry-matter yield, and biomass yield, genetic variance estimates were higher in the long-day length (rainy season) than in the shorter day length (summer season). Similarly, genetic variance estimates for yield and its component traits including panicle length, 1000-grain mass, and panicle grain number were marginally higher in the rainy season. However, for other traits including panicle diameter, panicle threshing percentage, and harvest index genetic variance estimates were marginally higher in the summer season. Estimates of variance component due to genotype × season (G × S) interaction were highly significant for all traits except for panicle threshing percentage. Genetic variance estimates for yield and its component traits were greater than estimates of G × S interactions.

FS progeny set mean performance of each of the five populations for phenology and grain and stover-yield related traits are presented in Supplementary file 3. The average number of days required to reach 75% of stigma emergence (FT) was earlier (54 days) in the rainy season than in the summer season (59 days). Among the populations, UCC 23 and “CO(Cu) 9” were later flowering in both seasons. The mean plant height was higher (216 cm) in the rainy season than in the summer (179 cm). In both seasons FS progenies of the advanced generation populations UCC 23 and CO(Cu) 9 recorded higher mean plant height. Trial mean panicle length was marginally higher in the summer season. However, similar mean panicle diameter values were observed in both seasons. About 10% improvement in panicle length was observed in advanced generation population UCC 23 compared to the base population (ICMP 87750), indicating that recurrent selection for this highly heritable grain yield component was effective.

Mean grain yield of CO(Cu) 9 (231 g/m^2^) was 11% higher than ICMP 87750 in the rainy season. However, ICMV 93752 recorded a significantly higher grain yield (175 g/m^2^) than “CO(Cu) 9” (161 g/m^2^) in the summer season trial. During the rainy season mean 1000-grain mass was higher (9.53 g) than that during the summer season trial (8.38 g). Similarly, mean panicle threshing percentage was also higher in the rainy season trial. Higher mean plant heights and numbers of tillers per plant in the rainy season produced higher mean stover dry-matter yield (322 g/m^2^) and biomass yield (619 g/m^2^) during the rainy season. In general, the mean values for grain yield, stover yield, and their component traits were higher in the early generation selection cycles, considerably decreased in mid-selection cycle, and increased further in the advanced selection cycles.

### 3.2. Genotypic Analysis

The integrated pearl millet genetic map of Qi et al. [11] was used as a reference for location and order of the 34 SSR markers used in the present study. These 34 markers were distributed across all 7 pearl millet linkage groups. A total of 159 alleles were detected for these 34 SSR loci. Among the 159 alleles observed, 49 were rare—having frequencies <5% across the 250 FS progenies. Frequency changes for rare alleles were not studied further due to the limited sample size used. The number of alleles per SSR locus ranged from 2 (*Xpsmp*2059 and *Xpsmp*2202) to 8 (*Xpsmp*2069) with an average of 4.68 alleles per locus (Supplementary file 2). The relative informativeness of each marker can be evaluated on the basis of its PIC value. PIC values of the 34 SSR loci used in this study of 250 FS progenies averaged 0.54 and ranged from 0.13 for *Xpsmp*2018 to 0.80 for *Xpsmp*2231. Allele frequency changes and Nei's unbiased genetic diversity at 34 SSR loci using full-sib progenies of “CO(Cu) 9” and their progenitor populations were studied as described earlier by Baskaran et al. [39].

### 3.3. AMOVA and Population Structure

**Figure 1:**
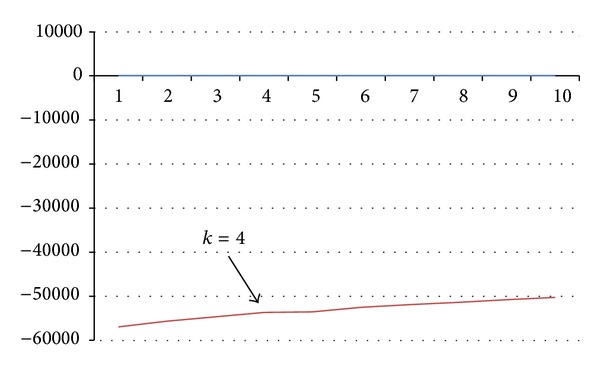
STRUCTURE results using 110 loci. Log probability data [LnP(*D*)] as function of *k* (number of groups) from the STRUCTURE run. The plateau of the graph at *k* = 4 indicates the minimum number of groups (subpopulations) possible in the panel.

**Figure 2:**
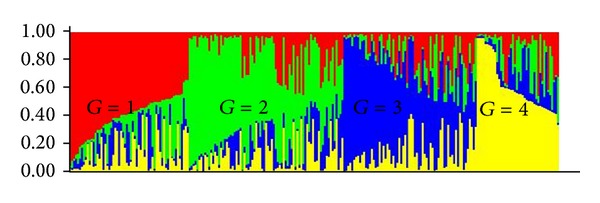
Estimated population structure of 250 FS progenies (*k* = 4). The *y*-axis is the group (subpopulation) membership, and the *x*-axis is the 250 individual FS genotypes. G (G1–G4) stands for group.

STRUCTURE software was run for *k* = 1–10 based on the distribution of 110 alleles at 34 SSR loci among 250 FS progenies. STRUCTURE simulation demonstrated that the *k* value showed a modest peak or flattening of the curve at *k* = 4, suggesting that four subpopulations could contain all individuals with greatest probability (Figure 1). Hence a *k* value of 4 groups (subpopulations) was selected to describe the genetic structure of the 250 FS progenies analyzed. Within and among group components of genetic variation were evaluated by AMOVA (Table 2). The results showed that FS progenies of within groups explained all of the observed genetic diversity (100%) and no differentiation was found among the groups (so the 250 FS progenies could be treated as a single nondifferentiated population, although that approach was not used for the analysis reported here). The population structure of essentially all of the FS progenies displayed partial membership to multiple groups, with few individual FS progenies exhibiting distinctive group identities (Table 3). Graphical representations of the 250 FS across these four groups (Figure 2 and Supplementary file 4) indicated that, among the 61 FS having group 1 as their largest component, 44.3% were from ICMP 87750, followed by UCC 23 (27.9%). Similarly, among the 79 FS having group 2 as their largest component, 35 (44.3%) were from CO(Cu) 9 followed by 21 (26.6%) from UCC 23; among the 68 FS having group 3 as their largest component, 37 (54.4%) were from ICMP 91751; and among the 42 FS having group 4 as their largest component, 27 (64.3%) were from ICMV 93752 (Table 3).

### 3.4. Association Analysis of Phenological Traits

The results of association analysis using GLM and MLM (*Q* + *K*) for the across-environment and two seasons (2005 and 2006) separately from this study are presented in Table 4 and Supplementary files 5a and 5b.

Seven SSR marker alleles were significantly associated with flowering time QTLs as detected by GLM (Table 4). Of these marker alleles *Xpsmp*2248_162 (LG6) had the strongest association followed by *Xpsmp*2248_166, *Xicmp*3027_202, and *Xpsmp*2088_136 as indicated by probability values. Significant association of *Xpsmp*2248_162 still could be detected after accounting for both population structure and relative kinship effects (*Q* + *K* model) in the across-environment and rainy season entry BLUPs (Tables 4 and 5). Moreover, *Xpsmp*2248_162 was associated with earlier flowering time. For plant height, as many as eight QTLs were detected segregating among the FS progenies by GLM, and *Xpsmp*2224_157 (LG7) was most strongly associated with plant height followed by *Xpsmp*2248_166 and *Xpsmp*2248_162 in the across-season data analysis as evidenced from probability values. Alleles *Xpsmp*2224_157 (LG7) and *Xpsmp*2085_175 (LG4) could also be detected by MLM in the across-season analysis. In addition to these marker alleles, *Xpsmp*2248_162 (LG6) was significantly associated with plant height as detected by MLM in the rainy season (Table 5). Thus, *Xpsmp*2248_162 was associated with QTL allele(s) conferring both reduced plant height and earlier flowering time.

### 3.5. Association Analysis of Grain and Stover-Yield Related Traits

Three SSR marker alleles (*Xpsmp*2077_136 (LG2), *Xpsmp*2233_260 (LG5), and *Xpsmp*2224_157 (LG7)) were associated with putative QTLs for panicle length as detected by GLM (Table 4). *Xpsmp*2077_136 was also significantly associated in the rainy season as detected by MLM (Table 5). *Xpsmp*2077_136 and *Xpsmp*2224_157 were positively correlated with panicle length. Marker alleles *Xicmp*3002_204 (LG6) and *Xpsmp*2248_164 (LG6) were significantly associated with genomic regions controlling panicle diameter variation, which was detected by GLM in the across-season analysis (Table 4). However, significant association of only the LG2 marker allele *Xpsmp*2201_364 could also detected by the mixed model and then only for the rainy season trial where this allele showed positive correlation with panicle diameter (Table 5 and Supplementary file 5a).

Association analysis across 250 FS progenies using GLM found that SSR allele *Xpsmp*2224_159 (LG7) was significantly associated with a QTL for grain yield, with this allele negatively correlated with that trait. Moreover, this association with *Xpsmp*2224_159 was also detected by MLM using the rainy season phenotype data set (Table 5), along with *Xpsmp*2237_230 (LG2) that could be detected by GLM (Supplementary file 5a). Marker allele *Xpsmp*2237_230 was positively correlated with grain yield.

Genomic regions controlling stover dry matter yield variation were detected on LG5, LG6, and LG7 in the across-season analysis using GLM, but no significant associations were detected by MLM with the across-season BLUPs for this trait (Table 4). However, *Xpsmp*2248_162 (LG6) and *Xicmp*3058_193 (LG6) were significantly associated with stover dry matter yield, as detected by MLM in the rainy and summer season, respectively (Table 5). *Xpsmp*2248_162 was negatively correlated with this trait, whereas *Xicmp*3058_193 was positively correlated (Supplementary files 5a and 5b). These two marker loci are separated by 13 cM [16], so they may detect the same QTL. For biomass yield, *Xpsmp*2224_159 (LG7) and *Xpsmp*2220_115 (LG2) were significantly associated as detected by GLM. *Xpsmp*2224_159 allele was negatively correlated with biomass yield and grain yield.

Two significant QTLs detected by GLM for panicle threshing percentage were associated with *Xpsmp*2227_196 (LG3) and *Xpsmp*2085_175 (LG4). The panicle threshing percentage QTL identified on LG4 was also detected by MLM using the across-season and summer season phenotype data sets (Tables 4 and 5). In addition, MLM detect associations of both *Xpsmp*2227_194 (LG3) and *Xpsmp*2227_196 (LG3), as well as *Xpsmp*2224_159 (LG7) using the rainy season data set, and *Xpsmp*2201_364 (LG2) using the summer season data set (Table 5). Two marker alleles *Xpsmp*2085_175 and *Xpsmp*2227_194 showed positive correlations with panicle threshing percentage (Table 4 and Supplementary file 5).

Three significant genomic regions were identified for harvest index by GLM in the across-season analysis (Table 4). Of the five marker alleles detected as associated with this trait by GLM, only *Xpsmp*2248_166 on LG6 was detected by MLM using the across-season and summer season phenotype data (Table 5). At least 5 possible QTLs for harvest index were detected by GLM using the rainy season data set (Supplementary file 5a). However, significant associations of only *Xpsmp*2027_229 and *Xpsmp*2224_159 (on LG7) and *Xpsmp*2246_261 (on LG1) were detected by MLM using the rainy season data. *Xpsmp*2224_159 and *Xpsmp*2248_166 were negatively correlated with harvest index, whereas *Xpsmp*2027_229 and *Xpsmp*2246_261 were positively correlated with this trait (Supplementary file 5a).

Two trait-specific alleles (*Xpsmp*2208_246 (LG5) and *Xpsmp*2277_242 (LG5)) were significantly associated with 1000-grain mass by GLM using the summer season phenotype data (Supplementary file 5b). These alleles were both negatively correlated with 1000-grain mass. However, these genomic regions were located on the same linkage group LG5. Significant association of *Xpsmp*2277_242 was also detected by MLM for this trait with this phenotype data set (Table 5). In contrast, with GLM using the rainy season phenotype data for this trait, only a weakly positive significant association with *Xpsmp*2030_112 (LG1) could be detected for this trait, but this could not be detected by MLM.

No significant marker-trait associations for panicle grain number or effective tiller number were detected by MLM, using the rainy season, summer season, or across-season BLUPs for these traits.

## 4. Discussion

In this study, G × S interactions were significant. However, genetic variance for yield and its component traits were greater than variance component estimates of the corresponding G × S interactions. This suggested that these grain and stover yield-related traits are pre-dominantly under genetic control. Significant G × E interactions can hinder genetic progress in breeding programs; in particular crossover-type G × E interaction makes it difficult to unambiguously select promising materials that consistently perform better across a wide range of environmental conditions [17]. Pearl millet is largely a quantitatively short day plant. However, mean performance of FS progenies showed about 5 days earlier flowering and relatively higher plant height in the rainy season (Table 1). It is suggested that warmer temperatures during early crop growth during the rainy season combined with the required level of photoperiod to induce floral initiation might have been responsible for this early flowering. In contrast, during the summer season field trial, initial temperatures were suboptimal for pearl millet seedling growth, which in turn delayed flowering despite short-day lengths that otherwise would be expected to induce early flowering. This was in agreement with the reports of Hellmers and Burton [40] and Yadav et al. [41].

Both panicle yield and grain yield means were greater in the rainy season trial. Higher mean values of their component traits in the rainy season contributed to these seasonal differences in grain and panicle yields. Mean grain yield of control entry “CO(Cu) 9” was 11% higher in the rainy season. However, earlier flowering mid-cycle population ICMV 93752 recorded a significantly higher grain yield than “CO(Cu) 9” in the summer season. This was probably a result of higher temperatures and greater evaporative demand during grain filling resulting in moderate drought stress in the summer season trial [42], despite weekly irrigation, with this stress having more adverse effects on grain filling of later-flowering FS progenies of UCC 23 and “CO(Cu) 9.” Support for this supposition is provided by the data on 1000-grain mass and panicle harvest index, which indicate that grain filling (especially of the later-flowering FS progenies) was compromised in the summer season trial [43]. Panicle grain number is an important component of the yield potential of a genotype under both severe stress and nonstress conditions [42]. The mean number of grains per panicle recorded was similar across the two seasons, indicating that drought stress was not severe in these trials during panicle development, flowering, and early grain-filling stages when grain number per panicle is determined. However, panicle harvest index and 1000-grain mass means were higher in the rainy season, suggesting that higher temperatures and restricted moisture availability may have forced maturity, resulting in incomplete grain filling during the summer season, and this would have had a more pronounced effect on late-flowering progenies [43].

The FS progenies exhibited higher mean stover dry-matter yield and biomass yield during the rainy season. This is likely due to their higher mean plant heights and numbers of effective tillers per plant in the rainy season [42]. FS progenies of ICMV 93752 recorded higher mean values for harvest index and were both shorter in height and earlier to flower than those of base population ICMP 87750 and the two most advanced populations in both seasons. This indicates not only that the initial two cycles of progeny-based selection in the rainy season at Patancheru (June sowings) were successful in increasing allele frequencies for reduced plant height and earlier flowering, but that the subsequent two cycles of mass selection at Coimbatore reversed the direction of selection for these phenological traits (to better meet the requirements for high yield potential in the shorter day-lengths of October sowing dates at Coimbatore). Increasing grain yield potential and yield stability have been major breeding objectives for improving yield performance. Increasing pearl millet grain yield through recurrent selection should be feasible due to its tremendous genetic variability [44] and the ease with which large numbers of test units and recombination units can be produced.

The graphical representations of the 250 FS across the four groups identified by the STRUCTURE analysis of the SSR marker data set (Supplementary file 4), suggest that the selection process leading to the development of CO(Cu) 9 began with the base population ICMP 87750 being comprised of individuals marker allele frequencies most like group 1. Selection for shorter plant height combined with medium maturity and long compact panicles to produce ICMP 91751 resulted in marker allele frequency changes leading to a predominance of individuals most like group 3. Subsequent selection for more uniform early maturity, longer panicles and higher grain yield to produce ICMV 93752 resulted in a further shift in marker allele frequencies most like group 4. The final two cycles of selection in the shorter day-length environment at Coimbatore resulted in substantial changes in allele frequencies, resulting in step-wise increase in individuals with marker allele frequencies most like group 2. It appears likely that selection against the few remaining individuals in CO(Cu) 9 having marker genotypes most like group 4 could marginally improve the performance (or at least the appearance and ease of maintenance) of this released variety.

AMOVA results in the present study indicated high genetic diversity (100%) within four subpopulations and that most of the genetic variation present in the population of 250 FS was attributable to heterozygosity of individual FS progenies at multiallelic loci. The initial composite population (ICMP 87750) used in this study was a random-mated half-sib composite bulk generated by intermating 62 S_2_ selections of the ICRISAT smut resistant composite (SRC) C_3_-cycle with 71 S_2_ selections of the ICRISAT intervarietal composite (IVC) C_7_-cycle, with reasonable numbers of selected progenies having been used to maintain genetic diversity during phenotypic recurrent selection cycles of its two progenitor populations, as well as during the four subsequent cycles of phenotypic recurrent selection that ended with the released improved population [CO(Cu) 9]. Therefore the level of genetic diversity within FS progenies used in this study was high, as expected. Although phenotypic recurrent selection generated patterns suggested by STRUCTURE as possibly representing four subpopulations among the 250 FS evaluated in this study, AMOVA indicated that these four subpopulations do not explain any of the marker variation detected within and among the 250 FS. Some previous studies in grass pea (*Lathyrus sativus* L.), peanut (*Arachis hypogaea* L.), soybean (*Glycine soja*), and peach (*Prunus persica*) have reported less or lower diversity between populations studied [45–49]. The model-based diversity and their significance are essential for population studies in plants. The model-based population structure of 250 FS progenies resulted in four groups (subpopulations) instead of five, although these groups accounted for essentially none of the observed SSR allelic variation among the 250 FS. The 50 FS progenies of UCC 23 could not establish separate groups but had their major shares in group 1 and group 2.

SSR marker allele on LG6 (*Xpsmp*2248_162) was significantly associated with flowering time (Tables 4 and 5) by both GLM and MLM. Position of the flowering time QTL detected in the present study (near *Xpsmp*2248) is similar to the one reported by Yadav et al. [17, 41]. However, all seven pearl millet linkage groups have been reported to contain genomic regions contributing to flowering time variation [17, 41, 50, 51]. Since the relatively strong negative correlation of *Xpsmp*2248_162 with flowering time appears to be real (i.e., significant association is detected by both GLM and MLM), it seems likely that the comparably strong positive correlation of *Xpsmp*2248_166 with flowering time is also likely to be real, even though it was not shown to be significant by MLM. Given that *Xpsmp*2248_162 had a negative correlation with flowering time, selection for this allele (and against allele *Xpsmp*2248_166) could be exploited to reduce flowering time, reduce plant height, and improve harvest index (see below). Alternatively, selection against *Xpsmp*2248_162 (and for allele *Xpsmp*2248_166) could be exploited to increase flowering time, increase plant height, and increase dry stover yield. The trait associations with these two alleles of this single SSR marker locus are those that we would expect of a QTL having substantial pleiotropic effects on these related traits.

Plant height influences both stover and biomass yields, and thereby harvest index. Positions of plant height QTLs detected on LG4, LG6, and LG7 were similar to those for flowering time, stover yield, and biomass yield QTLs reported by Yadav et al. [17, 41, 52]. Earlier, the pearl millet *d2* dwarfing gene was mapped on LG4 [53]. More recently it has been fine mapped and the likely underlying gene identified [54]. *Xpsmp*2248_162 (LG6) showed negative correlations with plant height, whereas *Xpsmp*2224_157 (LG7) and *Xpsmp*2085_175 (LG4) showed positive correlations with this trait. Hence, selection for *Xpsmp*2248_162 and against the alleles *Xpsmp*2224_157 and *Xpsmp*2085_175 could reduce plant height in the breeding population. Alternatively, selection for *Xpsmp*2224_157 and *Xpsmp*2085_175 and against allele *Xpsmp*2248_162 should result in increased plant height for production environments where higher stover yield is needed. Further, since the relatively strong negative correlation of *Xpsmp*2248_162 with plant appears to be real, as do the relatively strong positive correlations of *Xpsmp*2224_157 and *Xpsmp*2085_175 with this trait (i.e., significant associations are detected by both GLM and MLM), it seems likely that the comparably strong positive correlation of *Xpsmp*2248_166 and negative correlation of *Xpsmp*2224_159 with plant height are also likely to be real, even though they were not shown to be significant by MLM. Hence selection for or against these alternative alleles, as desired, is also likely to be effective in manipulating plant height, stover dry matter yield, and biomass yield.

SSR allele* Xpsmp*2077_136 on LG2 was associated with a putative QTL for panicle length (Table 5). Previous reports support the associations of genomic regions *Xpsmp*2077 [52] and *Xpsmp*2077 and *Xpsmp*2224 [55] with QTLs for grain yield. However, association of *Xpsmp*2224_157 with panicle length was detected only by GLM in this study. Both *Xpsmp*2077_236 and *Xpsmp*2224_157 were positively correlated with panicle length and grain yield and selection of progenies with these alleles could increase panicle length, which in turn should increase grain yield performance. Moreover, *Xpsmp*2224_157 was also associated with a plant height QTL (Table 4), and a second allele at this locus, *Xpsmp*2224_159, showed a significant association with grain yield and was negatively associated with both this trait and plant height in the rainy season environment. This indicates that potential for simultaneous improvement of grain and stover yield is possible if recurrent selection practiced in favor of *Xpsmp*2224_157 (and against *Xpsmp*2224_159). This is expected because of positive correlations between grain yield, stover, and biomass yield in pearl millet as described by Baskaran et al. [39]. SSR genomic region *Xpsmp*2201_364 mapped on LG2 was significantly associated with QTL controlling panicle diameter variation, but only in the rainy season (Table 5). Increasing the frequencies of favorable allele (*Xpsmp*2201_364) at this locus would likely be useful for the genetic improvement of panicle diameter in the populations used in this study. However, this same marker allele was significantly associated with panicle threshing percentage in the summer environment, where it showed a negative correlation with this trait. Previously, Yadav et al. [17, 41] reported this genomic region on LG2 as contributing to panicle number, grain yield, stover yield, panicle threshing percentage, and harvest index variation, and Bidinger et al. [55] reported QTLs for grain mass, grain yield, panicle threshing percentage and harvest index from this region.

For grain yield, significant associations of *Xpsmp*2224_159 (LG7) and *Xpsmp*2237_230 (LG2) were found by MLM and GLM, respectively (Table 5 and Supplementary file 5a), for the rainy season. The region on LG7 was also detected by GLM in the across-season analysis (Table 4), but no genomic regions associated with grain yield in the summer season were detected in this study. *Xpsmp*2224_159 was negatively correlated with grain yield (as well as with flowering time, plant height, and biomass yield) in the rainy season (Supplementary file 5a), whereas *Xpsmp*2237_230 was weakly positively correlated with grain yield in that environment. Bidinger et al. [55] identified a QTL for grain yield that mapped to LG7 (marker interval *Xpsmp*2224–*Xpsm*717) in an early-onset terminal drought stress environment. However, grain yield exhibited significant G × S interactions in the current study (Table 1), so the failure to detect the same QTLs for this trait in the rainy and the summer season trials was not surprising. This result agrees with findings of Veldboom and Lee [56] in maize, who reported differences in QTL detection in different environments, which were attributed to differential levels of expression of QTLs in those environments. In the current study, it appears possible that inadequate irrigation of the summer trial (discussed above) could have prevented detection of grain yield QTLs associated with later flowering in the summer environment.

In our study, allele *Xpsmp*2277_242 located on LG5 was significantly associated with 1000-grain mass variation in the summer season analysis (Table 5) and correlated with smaller grain mass (Supplementary file 5b). Interestingly, a second tightly linked marker allele (*Xpsmp*2208_246) showed a stronger negative correlation with 1000-grain mass in this season, but significance of the association was only detected by GLM. This QTL appears to be specific to 1000-grain mass, as associations of alleles of *Xpsmp*2277 and *Xpsmp*2208 could not be detected for other yield component traits. Grain mass, small grain mass with larger grain number, is an adaptive feature of pearl millet in arid regions where short grain-filling periods are common [57], whereas large grain mass is advantageous for improved rates of seedling emergence and plant stand [58].

The stover dry matter yield QTL detected on LG6 (by linked marker alleles *Xpsmp*2248_162 and *Xicmp*3058_193) coincided with QTLs for flowering time, plant height, biomass yield, and harvest index. Plant height is a significantly correlated trait of stover dry matter yield which explains these common associated genomic regions [39]. Significant G × S interaction was observed for stover dry matter yield (Table 1). Therefore, at least a portion of the QTLs detected for the trait in the present study were different for the rainy and the summer season trials (Supplementary files 5a and 5b). A QTL for biomass yield was associated with SSR marker allele *Xpsmp*2224_159 (LG7) in the rainy season (Table 5). This marker allele was negatively correlated with biomass yield and also comapped with QTLs for plant height and grain yield, as well as putative QTLs for flowering time, stover dry matter yield, panicle threshing percentage, and harvest index in this season (Supplementary file 5a). An alternate allele (*Xpsmp*2224_157) of this marker was positively and significantly associated with biomass component traits such as plant height, panicle length, and stover dry matter yield. Therefore, marker-assisted selection in favor of *Xpsmp*2224_157 (and against *Xpsmp*2224_159) could increase the biomass yield, and might also contribute favorably to grain yield, stover yield, panicle length, harvest index, and threshing percentage, all at the possible cost of increased flowering time in this population. Phenotypic correlations between these traits were described earlier by Baskaran et al. [39] for these populations.

Four significant QTLs (LG2, LG3, LG4, and LG7) were identified for panicle threshing percentage in different seasons (Table 5). Two marker alleles (*Xpsmp*2085_175 on LG4 and *Xpsmp*2227_194 on LG3) were positively correlated with this trait. Hence, selection for these alleles (and against *Xpsmp*2227_196, *Xpsmp*2224_159, and *Xpsmp*2201_364) can be expected to result in genetic improvement for this trait across the populations used in this study. Earlier, four QTLs for panicle harvest index were identified across moisture environments—each one on LG1, LG2, LG3, and LG6 [52, 55]. Moreover, one QTL detected for panicle threshing percentage in the present study on LG2 (*Xpsmp*2201) corresponds well with those previously detected by Bidinger et al. [55] using conventional QTL mapping in biparental populations (marker interval *Xpsmp*2059–*Xpsmp*2050). Panicle threshing percentage is a measure of pearl millet panicle compactness and has been used as a measure of tolerance to terminal drought, assessing the ability of a genotype to set and fill grains under limited moisture conditions [55].

Harvest index is a measure of the ability of a genotype to maximize partitioning of total biomass to grain yield. Four significant genomic regions (LG1, LG6, and two on LG7) were identified for harvest index (Table 5). SSR marker *Xpsmp*2248 (LG6) was also significantly associated with plant height and flowering time, as well as stover dry matter yield, whereas *Xpsmp*2224 (LG7) associated with grain yield QTL. As expected, *Xpsmp*2248_166 exhibited negative correlation with harvest index, since this allele was positively correlated with flowering time, plant height, and stover dry matter yield. These correlations were similar to those of phenotypic correlations of these populations [39]. Previously, Yadav et al. [41] identified a major QTL for pearl millet harvest index on LG6 and a minor QTL on LG2. Yadav et al. [52] and Bidinger et al. [55] identified QTLs for pearl millet harvest index on LG2, LG3, LG4, LG5, and LG7 for various grain-filling moisture environments. The LG6 (*Xpsmp*2248) and LG7 (*Xpsmp*2224) QTLs detected for harvest index in the present study appear to coincide with those previously reported by Yadav et al. [41, 52] and Bidinger et al. [55].

Conventional QTL mapping approaches identify genomic regions controlling traits having continuous variation by analysis of DNA marker profiles in structured populations derived from biparental crosses. An alternative approach, association mapping, has been proposed to identify only polymorphisms with extremely tight linkage to loci with significant phenotypic effects based on linkage disequilibrium in randomly mating populations [59]. With respect to association mapping populations, Breseghello and Sorrells [60] reported that synthetics offer a favorable balance of power and precision for association analysis to map quantitative traits with increasing resolution through cycles of intermating. In the current exploratory study, which was limited by the number of pearl millet SSR primer pairs detecting single codominant loci and did not exhibit unacceptable levels of template competition in PCR reactions, we have shown that this also appears to hold true for complex composite populations that are generated in recurrent selection programs. Because of this limitation, only a modest number of marker alleles could be used. Nonetheless, for highly heritable traits we were able to detect QTLs. The MLM approach was substantially more conservative that the GLM approach, as MLM typically detected significant associations with only one allele per marker locus, whereas GLM often detected significance for two marker alleles per locus, one having a positive correlation with the trait and the other a comparably strong negative correlation. In such cases, it appears that the MLM approach may be too conservative, and the biallelic associations detected by GLM are likely to be real ones that can be exploited in marker-assisted selection schemes.

As expected, correlated traits shared common genomic regions; this was especially apparent for associations of flowering time and plant height with yield and its component traits. The results suggest that simultaneous improvement of grain and stover yield could be achieved in pearl millet—provided that duration of moisture availability is not limiting—as there are some common genomic regions (and specific marker alleles) identified for these traits. Genomic regions detected by association analysis using CO(Cu) 9 and its progenitor populations were in agreement with the previous reports using different mapping populations in conventional QTL mapping studies [17, 41, 50–52, 55]. However, previous researchers detected larger numbers of QTLs for grain and stover yield-related traits than this study, demonstrating that larger numbers of markers are needed in association studies to provide better coverage across the entire nuclear genome, if they are to better than simple biparental populations—even in case of actively segregating breeding populations. The putative QTL regions identified in this study using the recurrent selection populations as well as in previous conventional mapping population studies should be of greatest importance in breeding programs.

## 5. Conclusions

Increasing the favorable alleles of significantly associated genomic regions would improve the grain and stover yield traits in future recurrent selection. The putative QTLs identified in this study, and significant favorable and unfavorable allelic associations detected, need to be validated before they can be recommended for applied use by pearl millet breeders. The first step proposed for this validation would be to attempt marker-based selection among three independent samples of 250 FS progenies across all five populations used in this study (50 FS progenies per population per sample) and assess performance of recombined bulks of the selected FS progenies in appropriate replicated multienvironment trials. Genomic regions identified in this study can provide an opportunity to develop a marker-assisted recurrent selection {MARS, Xie and Xu [61], which we prefer to refer to as marker-assisted population improvement (MAPI) to avoid confusion with the more common use of MARS coined by Charmet et al. [62]} approach to increase the rate of genetic gain during the development of improved pearl millet populations.

## Supplementary Material

Supplementary Material contains list of SSR markers used for genotyping the full-sib progenies of pearl millet populations, phenotype data generated from these populations, detailed population structure displayed by STRUCTURE program and association of SSR marker alleles with phenotypic traits during rainy season and summer season trial data sets.

## Figures and Tables

**Table 1:** Mean performance of 250 FS from CO (Cu) 9 and its immediate progenitor populations for phenology, grain, and stover-yield related traits in the rainy season 2005 and summer season 2006 at Patancheru, India.

Trait	Rainy season					Summer season					Across-season					
	Min.	Mean	Max.	*σ*_*g*_^2^	*h* ^2^	Min.	Mean	Max.	*σ*_*g*_^2^	*h* ^2^	Min.	Mean	Max.	*σ*_*g*_^2^	*σ*_*gs*_^2^	*h* ^2^
FT	47	54	61	24.16**	0.89	54	59	65	11.12**	0.83	51	56	62	26.99**	8.29**	0.71
PH	178	216	269	653.9**	0.87	155	179	217	378.5**	0.83	174	197	226	801.3**	244.4**	0.70
PL	22.2	27.5	33.9	17.14**	0.87	22.6	28.0	35.8	16.21**	0.84	23.0	27.8	33.7	29.91**	3.42**	0.83
PD	21.7	25.9	30.1	8.10**	0.81	20.4	25.1	29.9	9.98**	0.82	22.1	25.5	28.7	15.15**	2.93**	0.75
PY	197	297	379	4027**	0.69	118	242	333	3977**	0.69	224	270	321	5215**	2961**	0.52
GY	130	219	284	2716**	0.69	84	165	240	2072**	0.67	164	192	233	3003**	1870**	0.50
PTP	64.1	73.6	80.6	18.05**	0.65	57.5	68.2	74.8	24.32**	0.57	68.5	70.9	73.0	24.64**	17.76*	0.43
SDMY	176	322	488	8862**	0.82	166	268	397	4327**	0.75	223	295	375	9377**	4046**	0.62
BMY	413	619	817	20540**	0.76	328	511	687	10455**	0.71	460	566	666	19405**	11532**	0.53
TGM	7.05	9.53	12.12	2.85**	0.83	6.22	8.38	10.50	1.64**	0.75	7.55	8.96	10.52	3.40**	1.09**	0.67
PGN	1117	2054	3205	487189**	0.85	1229	2107	3287	338002**	0.73	1330	2081	2823	627865**	196027**	0.67
HI	27.5	35.3	41.0	17.95**	0.74	19.9	32.3	41.1	32.51**	0.70	29.5	33.8	38.0	32.61**	17.93**	0.54

*σ*_*g*_^2^: genotypic variance, *σ*_*gs*_^2^: genotype × season interaction variance, *h* ^2^: heritability; *significance at 0.05 level of probability, **significance at 0.01 level of probability; FT: flowering time (days), PH: plant height (cm), PL: panicle length (cm), PD: panicle diameter (mm), PY: panicle yield (g/m^2^), GY: grain yield (g/m^2^), PTP: panicle threshing percentage (%), SDMY: stover dry matter yield (g/m^2^), BMY: biomass yield (g/m^2^), TGM: 1000-grain mass (g), PGN: panicle grain number, and HI: harvest index (%).

**Table 2:** Analysis of genetic differentiation among genotypes of 250 FS progenies by AMOVA.

Source of variation	df	SS	MS	Est. var.	%	*P* value
Among groups	3	5.304	1.768	0.003	0	0.000
Within groups	246	392.30	1.595	1.595	100	0.001
Total	249	397.604		1.608	100	0.001

df: degrees of freedom, SS: sum of squares deviation, MS: mean squared deviation, Est. var.: estimates of variance components, and %: percentage of total variance contributed by each component.

**Table 3:** Significant divergence between groups (subpopulations) and average distances (expected heterozygosity) between FS progenies in the same groups.

Population groups	*F*_ST_	Heterozygosity	Number of FS genotypes	Components^†^
G1	0.1657	0.5022	61	27 (ICMP 87750), 4 (ICMP 91751), 5 (ICMV 93752), 17 (UCC 23) and 8 [CO (Cu) 9]
G2	0.1417	0.5066	79	6 (ICMP 87750), 3 (ICMP 91751), 14 (ICMV 93752), 21 (UCC 23) and 35 [CO (Cu) 9]
G3	0.1928	0.4925	68	11 (ICMP 87750), 37 (ICMP 91751), 4 (ICMV 93752), 10 (UCC 23) and 6 [CO (Cu) 9]
G4	0.2426	0.4617	42	6 (ICMP 87750), 6 (ICMP 91751), 27 (ICMV 93752), 2 (UCC 23) and 1 [CO (Cu) 9]

*F*_ST_: fixation index as measure of genetic differentiation and ^†^number of FS contributed from each pearl millet population to the group.

**Table 4:** Association of marker alleles with phenotypic traits using GLM and MLM (*Q* + *K*) models and the across-season BLUPs.

Trait	Locus	Linkage group	Correlation with phenotype	GLM Model		MLM (*Q* + *K*) Model	
				*F* ratio	−log 10 *P* value	*F* ratio	−log 10 *P* value
FT	*Xicmp*3027_202	5	0.28	4.86	3.53	1.58	ns
	*Xpsmp*2076_160	4	0.20	3.46	2.31	2.26	ns
	*Xpsmp*2077_136	2	−0.02	5.40	3.45	1.98	ns
	*Xpsmp*2088_136	2	−0.15	4.44	3.16	2.52	ns
	*Xpsmp*2227_194	3	0.18	3.19	2.08	1.94	ns
	*Xpsmp*2248_162	6	−0.30	8.59	5.78	3.50	2.08
	*Xpsmp*2248_166	6	0.29	6.44	4.22	2.07	ns

PH	*Xicmp*3027_202	5	0.27	4.23	2.98	1.46	ns
	*Xpsmp*2069_214	1	0.24	3.24	2.12	1.50	ns
	*Xpsmp*2077_136	2	−0.02	4.65	2.91	2.38	ns
	*Xpsmp*2085_175	4	0.21	3.95	2.40	3.68	2.21
	*Xpsmp*2088_136	2	−0.14	3.37	2.23	2.38	ns
	*Xpsmp*2224_157	7	0.27	7.26	4.82	5.54	3.56
	*Xpsmp*2224_159	7	−0.24	3.79	2.28	2.12	ns
	*Xpsmp*2233_256	5	0.17	3.96	2.75	1.81	ns
	*Xpsmp*2233_260	5	−0.06	3.30	2.18	2.08	ns
	*Xpsmp*2233_262	5	−0.18	5.58	3.59	2.43	ns
	*Xpsmp*2248_162	6	−0.30	6.17	4.02	2.80	ns
	*Xpsmp*2248_166	6	0.30	6.43	4.21	2.70	ns

PL	*Xpsmp*2077_136	2	0.09	4.21	2.58	2.30	ns
	*Xpsmp*2224_157	7	0.12	3.46	2.04	3.27	ns
	*Xpsmp*2233_260	5	−0.13	4.00	2.78	2.88	ns

PD	*Xicmp*3002_204	6	0.22	3.44	2.30	2.56	ns
	*Xpsmp*2248_164	6	0.01	4.76	2.03	3.30	ns

GY	*Xpsmp*2224_159	7	−0.25	4.20	2.58	3.16	ns

SDMY	*Xpsmp*2220_115	5	0.14	3.23	2.11	1.84	ns
	*Xpsmp*2224_157	7	0.21	4.21	2.59	2.82	ns
	*Xpsmp*2233_262	5	−0.18	3.97	2.41	2.90	ns
	*Xpsmp*2248_162	6	−0.23	3.90	2.36	2.56	ns
	*Xpsmp*2248_166	6	0.21	3.43	2.03	1.64	ns

BMY	*Xpsmp*2220_115	5	0.15	3.09	2.00	2.10	ns
	*Xpsmp*2224_159	7	−0.22	3.64	2.18	2.09	ns

PTP	*Xpsmp*2085*_*175	4	0.05	4.10	2.51	3.95	2.40
	*Xpsmp*2227_196	3	−0.20	3.70	2.52	2.95	ns

HI	*Xicmp*3027_200	5	0.26	4.39	3.12	2.51	ns
	*Xicmp*3027_202	5	−0.22	3.85	2.65	1.95	ns
	*Xpsmp*2086_116	4	0.16	3.14	2.04	1.99	ns
	*Xpsmp*2248_162	6	0.23	4.98	3.15	3.08	ns
	*Xpsmp*2248_166	6	−0.25	6.59	4.33	4.37	2.70

FT: flowering time, PH: plant height, PL: panicle length, PD: panicle diameter, GY: grain yield, SDMY: stover dry matter yield, BMY: biomass yield, PTP: panicle threshing percentage, and HI: harvest index.

**Table 5:** Lists of SSR marker alleles significantly associated with phenotypic traits using the *Q* + *K* model and the individual season BLUPs.

Trait	Across-seasons	Rainy season 2005	Summer season 2006
FT	*Xpsmp*2248_162 (LG6)	*Xpsmp*2248_162 (LG6)	—

PH	*Xpsmp*2085_175 (LG4) *Xpsmp*2224_157 (LG7)	*Xpsmp*2085_175 (LG4) *Xpsmp*2248_162 (LG6) *Xpsmp*2224_157 (LG7)	*Xpsmp*2224_157 (LG7)

PL	—	*Xpsmp*2077_136 (LG2)	—

PD	—	*Xpsmp*2201_364 (LG2)	—

GY	—	*Xpsmp*2224_159 (LG7)	—

SDMY	—	*Xpsmp*2248_162 (LG6)	*Xicmp*3058_193 (LG6)

BMY	—	*Xpsmp*2224_159 (LG7)	—

PTP	*Xpsmp*2085*_*175 (LG4)	*Xpsmp*2227_194 (LG3) *Xpsmp*2227_196 (LG3) *Xpsmp*2224_159 (LG7)	*Xpsmp*2201_364 (LG2) *Xpsmp*2085_175 (LG4)

TGM	—	—	*Xpsmp*2277_242 (LG5)

HI	*Xpsmp*2248_166 (LG6)	*Xpsmp*2246_261 (LG1) *Xpsmp*2027_229 (LG7) *Xpsmp*2224_159 (LG7)	*Xpsmp*2248_166 (LG6)

FT: flowering time, PH: plant height, PL: panicle length, PD: panicle diameter, GY: grain yield, SDMY: stover dry matter yield, BMY: biomass yield, PTP: panicle threshing percentage, TGM: 1000-grain mass, HI: harvest index, and LG: linkage group.
