# Supplementary material for: Association Analysis of SSR Markers with Phenology, Grain, and Stover-Yield Related Traits in Pearl Millet (Pennisetum glaucum (L.) R. Br.)
Source: ScientificWorldJournal. 2014 Jan 2;2014:562327. doi: 10.1155/2014/562327 (PMC3910278; doi:10.1155/2014/562327)
Supplement: Supplementary file 1 — Supplementary Material contains list of SSR markers used for genotyping the full-sib progenies of pearl millet populations, phenotype data generated from these populations, detailed population structure displayed by STRUCTURE program and association of SSR marker alleles with phenotypic traits during rainy season and summer season trial data sets. [file 562327.f1.docx]

Supplementary file 1: List of 34 SSR loci genotyped for the five pearl millet FS progeny sets.

| SSR locus | LG | No. of alleles | | Observed PIC value | References |
| --- | --- | --- | --- | --- | --- |
|  |  | Common | Rare |  |  |
| *Xpsmp*2030 | 1 | 4 | 2 | 0.60 | Qi et al. (2004) |
| *Xpsmp*2069 | 1 | 4 | 4 | 0.59 | Qi et al. (2004); Rajaram et al. (2013) |
| *Xpsmp*2090 | 1 | 4 | 3 | 0.65 | Qi et al. (2004) |
| *Xpsmp*2246 | 1 | 3 | 0 | 0.58 | Allouis et al. (2001); Qi et al. (2004) |
| *Xicmp*3017 | 1 | 3 | 0 | 0.66 | Senthilvel et al. (2004, 2008); Rajaram et al. (2013) |
| *Xicmp*3032 | 1 | 3 | 0 | 0.66 | Senthilvel et al. (2004, 2008); Rajaram et al. (2013) |
| *Xpsmp*2059 | 2 | 2 | 0 | 0.22 | Qi et al. (2004); Rajaram et al. (2013) |
| *Xpsmp*2072 | 2 | 5 | 1 | 0.77 | Qi et al. (2004); Rajaram et al. (2013) |
| *Xpsmp*2077 | 2 | 4 | 3 | 0.42 | Qi et al. (2004) |
| *Xpsmp*2088 | 2 | 3 | 4 | 0.66 | Qi et al. (2004); Rajaram et al. (2013) |
| *Xpsmp*2201 | 2 | 2 | 1 | 0.25 | Qi et al. (2001); Qi et al. (2004) |
| *Xpsmp*2211 | 2 | 2 | 0 | 0.33 | Qi et al. (2001); Qi et al. (2004) |
| *Xpsmp*2225 | 2 | 3 | 1 | 0.59 | Qi et al. (2001); Qi et al. (2004) |
| *Xpsmp*2231 | 2 | 6 | 1 | 0.80 | Allouis et al. (2001); Qi et al. (2004); Rajaram et al. (2013) |
| *Xpsmp*2237 | 2 | 2 | 2 | 0.31 | Allouis et al. (2001); Qi et al. (2004); Rajaram et al. (2013) |
| *Xpsmp*2227 | 3 | 2 | 2 | 0.40 | Allouis et al. (2001); Qi et al. (2004); Rajaram et al. (2013) |
| *Xpsmp*2008 | 4 | 4 | 0 | 0.72 | Qi et al. (2004); Rajaram et al. (2013) |
| *Xpsmp*2076 | 4 | 2 | 4 | 0.58 | Qi et al. (2004); Rajaram et al. (2013) |
| *Xpsmp*2085 | 4 | 4 | 1 | 0.66 | Qi et al. (2004); Rajaram et al. (2013) |
| *Xpsmp*2086 | 4 | 3 | 3 | 0.68 | Qi et al. (2004); Rajaram et al. (2013) |
| *Xpsmp*2202 | 5 | 2 | 0 | 0.49 | Qi et al. (2001); Qi et al. (2004) |
| *Xpsmp*2208 | 5 | 3 | 1 | 0.25 | Qi et al. (2001); Rajaram et al. (2013) |
| *Xpsmp*2220 | 5 | 4 | 3 | 0.72 | Qi et al. (2001); Qi et al. (2004) |
| *Xpsmp*2233 | 5 | 4 | 1 | 0.57 | Allouis et al. (2001); Qi et al. (2004); Rajaram et al. (2013) |
| *Xpsmp*2277 | 5 | 4 | 1 | 0.71 | Bertin (unpublished); Rajaram et al. (2013) |
| *Xicmp*3027 | 5 | 2 | 3 | 0.59 | Senthilvel et al. (2004, 2008); Rajaram et al. (2013) |
| *Xpsmp*2018 | 6 | 1 | 2 | 0.13 | Qi et al. (2004) |
| *Xpsmp*2248 | 6 | 3 | 1 | 0.52 | Allouis et al. (2001); Qi et al. (2004); Rajaram et al. (2013) |
| *Xpsmp*2275 | 6 | 4 | 0 | 0.53 | Bertin (unpublished); Rajaram et al. (2013) |
| *Xicmp*3002 | 6 | 2 | 2 | 0.53 | Senthilvel et al. (2004, 2008); Rajaram et al. (2013) |
| *Xicmp*3058 | 6 | 3 | 1 | 0.49 | Senthilvel et al. (2004, 2008); Rajaram et al. (2013) |
| *Xpsmp*2027 | 7 | 5 | 1 | 0.70 | Qi et al. (2004) |
| *Xpsmp*2087 | 7 | 4 | 0 | 0.61 | Qi et al. (2004); Rajaram et al. (2013) |
| *Xpsmp*2224 | 7 | 4 | 1 | 0.54 | Qi et al. (2001); Qi et al. (2004); Rajaram et al. (2013) |

LG = Linkage group; PIC = Polymorphism information content

Supplementary file 2: Expected genetic constitution of a full-sib progeny in relation to its parental genetic constitution.

| Parent 1 | Parent 2 | Expected genetic constitution of full-sib progeny |
| --- | --- | --- |
| a and b | c and d | a,b,c,d – four alleles, each with allelic frequencies of 0.25 |
| a and b or a and c | c and c or b and c | a,b,c,c – three alleles, two with allelic frequencies of 0.25 and one with an allelic frequency of 0.50 |
| a and a or a and b | b and b or a and b | a,a,b,b – two alleles, each with allelic frequencies of 0.50 |
| a and a | a and b | a,a,a,b – two alleles, one with an allelic frequency of 0.75 and the other with an allelic frequency of 0.25 |
| a and a | a and a | a,a,a,a – only one allele with an allelic frequency of 1.00 |

Supplementary file 3: Summary of statistical analysis of phenotype data

| **Trait** | **Population** | **Rainy season 2005** | | | | | |  | **Summer season 2006** | | | | | |  | **Across-seasons** | | | | | | | |
| --- | --- | --- | --- | --- | --- | --- | --- | --- | --- | --- | --- | --- | --- | --- | --- | --- | --- | --- | --- | --- | --- | --- | --- |
|  |  | **Min** | **Mean** | **Max** | **σ^2^g** | **SE**  **(σ^2^g)** | ***h^2^*** |  | **Min** | **Mean** | **Max** | **σ^2^g** | **SE**  **(σ^2^g)** | ***h^2^*** |  | **Min** | **Mean** | **Max** | **σ^2^g** | **SE**  **(σ^2^g)** | **σ^2^gs** | **SE**  **(σ^2^gs)** | ***h^2^*** |
| **FT** | **ICMP 87750** | 48 | 54 | 59 | 16.49** | 0.57 | 0.82 |  | 55 | 59 | 63 | 8.85** | 0.42 | 0.79 |  | 52 | 57 | 60 | 18.93** | 0.61 | 6.41** | 0.70 | 0.67 |
|  | **ICMP 91751** | 48 | 52 | 57 | 13.37** | 0.51 | 0.78 |  | 54 | 58 | 62 | 7.11** | 0.37 | 0.77 |  | 52 | 55 | 58 | 13.27** | 0.51 | 7.21** | 0.63 | 0.57 |
|  | **ICMV 93752** | 47 | 52 | 57 | 17.27** | 0.58 | 0.88 |  | 54 | 57 | 63 | 10.36** | 0.45 | 0.83 |  | 51 | 55 | 60 | 20.39** | 0.63 | 7.23** | 0.74 | 0.68 |
|  | **UCC 23** | 50 | 56 | 60 | 18.65** | 0.60 | 0.87 |  | 56 | 60 | 65 | 12.41** | 0.49 | 0.84 |  | 54 | 58 | 62 | 21.24** | 0.65 | 9.82** | 0.78 | 0.63 |
|  | **CO (Cu) 9** | 49 | 55 | 61 | 20.38** | 0.63 | 0.88 |  | 55 | 59 | 63 | 6.33** | 0.35 | 0.74 |  | 54 | 57 | 61 | 19.56** | 0.62 | 7.13** | 0.72 | 0.67 |
|  | **Overall** | 47 | 54 | 61 | 24.16** | 0.69 | 0.89 |  | 54 | 59 | 65 | 11.12** | 0.47 | 0.83 |  | 51 | 56 | 62 | 26.99** | 0.73 | 8.29** | 0.83 | 0.71 |
|  |  |  |  |  |  |  |  |  |  |  |  |  |  |  |  |  |  |  |  |  |  |  |  |
| **PH** | **ICMP 87750** | 185 | 215 | 239 | 427.9** | 2.9 | 0.78 |  | 160 | 179 | 197 | 179.0** | 1.9 | 0.65 |  | 176 | 197 | 216 | 441.2** | 2.9 | 165.7* | 3.4 | 0.62 |
|  | **ICMP 91751** | 180 | 205 | 228 | 445.8** | 3.0 | 0.83 |  | 156 | 174 | 217 | 467.1** | 3.0 | 0.90 |  | 174 | 190 | 212 | 687.8** | 3.7 | 228.6** | 4.2 | 0.69 |
|  | **ICMV 93752** | 187 | 207 | 235 | 324.7** | 2.5 | 0.82 |  | 157 | 173 | 200 | 300.1** | 2.4 | 0.82 |  | 177 | 191 | 210 | 464.0** | 3.0 | 160.8** | 3.5 | 0.66 |
|  | **UCC 23** | 198 | 225 | 269 | 573.7** | 3.4 | 0.85 |  | 162 | 185 | 206 | 389.2** | 2.8 | 0.80 |  | 188 | 204 | 226 | 615.3** | 3.5 | 341.1** | 4.3 | 0.58 |
|  | **CO (Cu) 9** | 178 | 223 | 248 | 586.5** | 3.4 | 0.83 |  | 155 | 181 | 205 | 318.3** | 2.5 | 0.80 |  | 174 | 201 | 219 | 805.5** | 4.0 | 215.1** | 4.5 | 0.72 |
|  | **Overall** | 178 | 216 | 269 | 653.9** | 3.6 | 0.87 |  | 155 | 179 | 217 | 378.5** | 2.7 | 0.83 |  | 174 | 197 | 226 | 801.3** | 4.0 | 244.4** | 4.5 | 0.70 |
|  |  |  |  |  |  |  |  |  |  |  |  |  |  |  |  |  |  |  |  |  |  |  |  |
| **PL** | **ICMP 87750** | 22.3 | 26.3 | 30.7 | 14.46** | 0.53 | 0.84 |  | 22.6 | 26.9 | 31.2 | 13.40** | 0.51 | 0.83 |  | 23.0 | 26.7 | 30.1 | 24.14** | 0.69 | 3.71^ns^ | 0.74 | 0.79 |
|  | **ICMP 91751** | 22.2 | 26.9 | 31.9 | 12.55** | 0.50 | 0.83 |  | 23.5 | 27.4 | 32.4 | 12.84** | 0.50 | 0.84 |  | 23.3 | 27.2 | 31.7 | 22.55** | 0.66 | 2.84^ns^ | 0.71 | 0.81 |
|  | **ICMV 93752** | 22.7 | 26.9 | 33.3 | 15.32** | 0.55 | 0.88 |  | 23.1 | 27.3 | 31.7 | 11.30** | 0.47 | 0.76 |  | 23.6 | 27.2 | 31.9 | 24.24** | 0.69 | 2.38^ns^ | 0.72 | 0.82 |
|  | **UCC 23** | 24.3 | 29.0 | 33.9 | 15.26** | 0.55 | 0.88 |  | 24.5 | 29.5 | 35.8 | 15.20** | 0.55 | 0.82 |  | 25.2 | 29.1 | 33.7 | 25.89** | 0.71 | 4.55** | 0.77 | 0.78 |
|  | **CO (Cu) 9** | 23.3 | 27.9 | 33.5 | 17.98** | 0.59 | 0.88 |  | 23.4 | 28.4 | 35.0 | 16.71** | 0.57 | 0.84 |  | 23.9 | 28.1 | 33.7 | 30.99** | 0.78 | 3.70^ns^ | 0.82 | 0.82 |
|  | **Overall** | 22.2 | 27.5 | 33.9 | 17.14** | 0.58 | 0.87 |  | 22.6 | 28.0 | 35.8 | 16.21** | 0.56 | 0.84 |  | 23.0 | 27.8 | 33.7 | 29.91** | 0.77 | 3.42** | 0.81 | 0.83 |
|  |  |  |  |  |  |  |  |  |  |  |  |  |  |  |  |  |  |  |  |  |  |  |  |
| **PD** | **ICMP 87750** | 22.1 | 25.8 | 29.3 | 7.30** | 0.38 | 0.83 |  | 22.0 | 25.4 | 28.9 | 8.38** | 0.41 | 0.77 |  | 22.5 | 25.6 | 28.3 | 13.99** | 0.52 | 1.67^ns^ | 0.55 | 0.79 |
|  | **ICMP 91751** | 21.7 | 25.0 | 28.1 | 7.35** | 0.38 | 0.79 |  | 21.6 | 24.3 | 27.6 | 7.42** | 0.38 | 0.77 |  | 22.5 | 24.8 | 27.5 | 11.90** | 0.48 | 2.87^ns^ | 0.54 | 0.71 |
|  | **ICMV 93752** | 22.4 | 25.3 | 28.8 | 6.24** | 0.35 | 0.79 |  | 20.4 | 23.8 | 28.0 | 8.89** | 0.42 | 0.81 |  | 22.1 | 24.7 | 28.0 | 12.56** | 0.50 | 2.58^ns^ | 0.54 | 0.74 |
|  | **UCC 23** | 23.0 | 26.2 | 30.1 | 7.19** | 0.38 | 0.79 |  | 22.0 | 25.5 | 29.3 | 9.95** | 0.44 | 0.83 |  | 23.6 | 25.8 | 28.7 | 13.98** | 0.52 | 3.17** | 0.58 | 0.73 |
|  | **CO (Cu) 9** | 23.2 | 26.8 | 29.5 | 7.95** | 0.39 | 0.77 |  | 22.5 | 25.9 | 29.9 | 7.78** | 0.39 | 0.77 |  | 23.6 | 26.2 | 28.5 | 12.58** | 0.50 | 3.12^ns^ | 0.55 | 0.70 |
|  | **Overall** | 21.7 | 25.9 | 30.1 | 8.10** | 0.40 | 0.81 |  | 20.4 | 25.1 | 29.9 | 9.98** | 0.44 | 0.82 |  | 22.1 | 25.5 | 28.7 | 15.15** | 0.55 | 2.93** | 0.60 | 0.75 |
|  |  |  |  |  |  |  |  |  |  |  |  |  |  |  |  |  |  |  |  |  |  |  |  |
| **HC** | **ICMP 87750** | 7 | 11 | 15 | 9.38** | 0.43 | 0.75 |  | 5 | 9 | 13 | 7.64** | 0.39 | 0.69 |  | 8 | 10 | 13 | 12.15** | 0.49 | 4.90* | 0.58 | 0.60 |
|  | **ICMP 91751** | 10 | 12 | 15 | 4.23ns | 0.29 | 0.53 |  | 7 | 10 | 14 | 6.80** | 0.37 | 0.70 |  | 9 | 11 | 13 | 7.03** | 0.37 | 3.96ns | 0.46 | 0.49 |
|  | **ICMV 93752** | 9 | 12 | 15 | 4.06* | 0.28 | 0.61 |  | 6 | 11 | 15 | 7.19ns | 0.38 | 0.57 |  | 9 | 11 | 13 | 6.96** | 0.37 | 4.26ns | 0.47 | 0.45 |
|  | **UCC 23** | 8 | 11 | 14 | 5.46** | 0.33 | 0.68 |  | 5 | 9 | 12 | 6.64** | 0.36 | 0.69 |  | 8 | 10 | 12 | 8.63** | 0.41 | 3.46ns | 0.49 | 0.58 |
|  | **CO (Cu) 9** | 7 | 11 | 14 | 5.87** | 0.34 | 0.69 |  | 5 | 9 | 12 | 5.89** | 0.34 | 0.66 |  | 9 | 10 | 12 | 9.27** | 0.43 | 3.44ns | 0.50 | 0.60 |
|  | **Overall** | 7 | 11 | 15 | 6.32** | 0.35 | 0.68 |  | 5 | 10 | 15 | 8.08** | 0.40 | 0.69 |  | 8 | 11 | 13 | 10.67** | 0.46 | 3.96* | 0.54 | 0.60 |

| **Trait** | **Population** | **Rainy season 2005** | | | | | |  | **Summer season 2006** | | | | | |  | **Across-seasons** | | | | | | | |
| --- | --- | --- | --- | --- | --- | --- | --- | --- | --- | --- | --- | --- | --- | --- | --- | --- | --- | --- | --- | --- | --- | --- | --- |
|  |  | **Min** | **Mean** | **Max** | **σ^2^g** | **SE**  **(σ^2^g)** | ***h^2^*** |  | **Min** | **Mean** | **Max** | **σ^2^g** | **SE**  **(σ^2^g)** | ***h^2^*** |  | **Min** | **Mean** | **Max** | **σ^2^g** | **SE**  **(σ^2^g)** | **σ^2^gs** | **SE**  **(σ^2^gs)** | ***h^2^*** |
| **ET** | **ICMP 87750** | 1.10 | 1.59 | 2.20 | 0.22** | 0.07 | 0.75 |  | 1.00 | 1.34 | 2.15 | 0.20** | 0.06 | 0.76 |  | 1.19 | 1.43 | 1.75 | 0.32** | 0.08 | 0.10^ns^ | 0.09 | 0.65 |
|  | **ICMP 91751** | 1.05 | 1.50 | 2.07 | 0.14** | 0.05 | 0.71 |  | 0.96 | 1.30 | 2.49 | 0.24** | 0.07 | 0.85 |  | 1.19 | 1.39 | 1.93 | 0.30** | 0.08 | 0.07^ns^ | 0.09 | 0.70 |
|  | **ICMV 93752** | 1.20 | 1.50 | 1.86 | 0.06** | 0.03 | 0.44 |  | 1.00 | 1.36 | 2.03 | 0.15** | 0.05 | 0.71 |  | 1.24 | 1.42 | 1.68 | 0.13** | 0.05 | 0.08^ns^ | 0.06 | 0.46 |
|  | **UCC 23** | 1.17 | 1.43 | 1.90 | 0.09** | 0.04 | 0.63 |  | 0.86 | 1.18 | 1.83 | 0.09** | 0.04 | 0.67 |  | 1.16 | 1.33 | 1.66 | 0.11** | 0.05 | 0.06^ns^ | 0.06 | 0.51 |
|  | **CO (Cu) 9** | 1.14 | 1.48 | 1.98 | 0.13** | 0.05 | 0.69 |  | 0.86 | 1.18 | 1.67 | 0.08** | 0.04 | 0.64 |  | 1.13 | 1.34 | 1.57 | 0.14** | 0.05 | 0.07^ns^ | 0.06 | 0.53 |
|  | **Overall** | 1.05 | 1.50 | 2.20 | 0.13** | 0.05 | 0.68 |  | 0.86 | 1.27 | 2.49 | 0.17** | 0.06 | 0.76 |  | 1.13 | 1.38 | 1.93 | 0.22** | 0.07 | 0.08** | 0.08 | 0.62 |
|  |  |  |  |  |  |  |  |  |  |  |  |  |  |  |  |  |  |  |  |  |  |  |  |
| **PY** | **ICMP 87750** | 206 | 283 | 359 | 4486** | 9.4 | 0.69 |  | 169 | 232 | 318 | 4225** | 9.1 | 0.69 |  | 227 | 263 | 300 | 5967** | 10.8 | 2746^ns^ | 13.1 | 0.56 |
|  | **ICMP 91751** | 197 | 283 | 375 | 4244** | 9.1 | 0.71 |  | 144 | 244 | 333 | 3295** | 8.0 | 0.65 |  | 237 | 267 | 321 | 4498** | 9.4 | 3046** | 12.2 | 0.49 |
|  | **ICMV 93752** | 222 | 293 | 356 | 3111** | 7.8 | 0.70 |  | 179 | 256 | 332 | 2911** | 7.6 | 0.63 |  | 247 | 273 | 297 | 3256** | 8.0 | 2762** | 10.9 | 0.43 |
|  | **UCC 23** | 209 | 304 | 360 | 2851** | 7.5 | 0.65 |  | 152 | 237 | 315 | 4353** | 9.2 | 0.67 |  | 224 | 271 | 305 | 4473** | 9.4 | 2753* | 11.9 | 0.49 |
|  | **CO (Cu) 9** | 232 | 311 | 379 | 4024** | 8.9 | 0.64 |  | 118 | 237 | 318 | 5014** | 9.9 | 0.77 |  | 235 | 273 | 310 | 7713** | 12.3 | 2338^ns^ | 14.0 | 0.65 |
|  | **Overall** | 197 | 297 | 379 | 4027** | 8.9 | 0.69 |  | 118 | 242 | 333 | 3977** | 8.8 | 0.69 |  | 224 | 270 | 321 | 5215** | 10.1 | 2961** | 12.7 | 0.52 |
|  |  |  |  |  |  |  |  |  |  |  |  |  |  |  |  |  |  |  |  |  |  |  |  |
| **GY** | **ICMP 87750** | 142 | 207 | 277 | 2940** | 7.6 | 0.68 |  | 105 | 159 | 218 | 2221** | 6.6 | 0.68 |  | 164 | 188 | 222 | 3481** | 8.3 | 1696^ns^ | 10.1 | 0.54 |
|  | **ICMP 91751** | 130 | 208 | 283 | 3044** | 7.7 | 0.72 |  | 96 | 166 | 240 | 1879** | 6.1 | 0.64 |  | 166 | 190 | 233 | 2728** | 7.3 | 2187** | 9.8 | 0.45 |
|  | **ICMV 93752** | 161 | 215 | 277 | 2054** | 6.3 | 0.68 |  | 114 | 175 | 234 | 1603** | 5.6 | 0.61 |  | 173 | 194 | 211 | 1956** | 6.2 | 1701** | 8.5 | 0.42 |
|  | **UCC 23** | 159 | 225 | 273 | 1909** | 6.1 | 0.63 |  | 105 | 162 | 218 | 2213** | 6.6 | 0.65 |  | 164 | 193 | 218 | 2636** | 7.2 | 1518^ns^ | 9.0 | 0.50 |
|  | **CO (Cu) 9** | 169 | 231 | 284 | 2603** | 7.1 | 0.64 |  | 84 | 161 | 220 | 2431** | 6.9 | 0.72 |  | 170 | 195 | 221 | 4056** | 8.9 | 1453^ns^ | 10.4 | 0.60 |
|  | **Overall** | 130 | 219 | 284 | 2716** | 7.3 | 0.69 |  | 84 | 165 | 240 | 2072** | 6.4 | 0.67 |  | 164 | 192 | 233 | 3003** | 7.7 | 1870** | 9.8 | 0.50 |

| **PTP** | **ICMP 87750** | 67.1 | 73.3 | 77.5 | 14.75* | 0.54 | 0.61 |  | 57.5 | 68.5 | 74.8 | 33.82** | 0.81 | 0.68 |  | 68.9 | 70.9 | 73.0 | 23.87** | 0.68 | 24.17** | 0.97 | 0.39 |
| --- | --- | --- | --- | --- | --- | --- | --- | --- | --- | --- | --- | --- | --- | --- | --- | --- | --- | --- | --- | --- | --- | --- | --- |
|  | **ICMP 91751** | 68.4 | 73.1 | 80.6 | 27.12** | 0.73 | 0.71 |  | 57.7 | 67.8 | 73.2 | 25.10^ns^ | 0.70 | 0.53 |  | 69.0 | 70.8 | 72.6 | 31.34** | 0.78 | 20.72^ns^ | 1.01 | 0.44 |
|  | **ICMV 93752** | 67.5 | 73.3 | 77.7 | 15.21** | 0.55 | 0.59 |  | 59.9 | 67.9 | 73.4 | 26.05* | 0.71 | 0.61 |  | 69.3 | 70.8 | 72.7 | 25.67** | 0.71 | 16.13^ns^ | 0.91 | 0.45 |
|  | **UCC 23** | 69.8 | 73.6 | 79.2 | 12.47^ns^ | 0.49 | 0.58 |  | 62.3 | 68.3 | 73.1 | 18.58^ns^ | 0.60 | 0.56 |  | 69.6 | 70.9 | 72.5 | 18.76* | 0.61 | 12.60^ns^ | 0.78 | 0.43 |
|  | **CO (Cu) 9** | 64.1 | 73.9 | 79.1 | 21.71** | 0.65 | 0.72 |  | 63.4 | 68.2 | 73.4 | 18.99^ns^ | 0.61 | 0.48 |  | 68.5 | 71.0 | 72.6 | 26.31** | 0.72 | 14.54^ns^ | 0.89 | 0.47 |
|  | **Overall** | 64.1 | 73.6 | 80.6 | 18.05** | 0.59 | 0.65 |  | 57.5 | 68.2 | 74.8 | 24.32** | 0.69 | 0.57 |  | 68.5 | 70.9 | 73.0 | 24.64** | 0.70 | 17.76* | 0.91 | 0.43 |
|  |  |  |  |  |  |  |  |  |  |  |  |  |  |  |  |  |  |  |  |  |  |  |  |
| **SDMY** | **ICMP 87750** | 208 | 310 | 488 | 9359** | 13.5 | 0.78 |  | 185 | 269 | 338 | 3613** | 8.4 | 0.71 |  | 223 | 291 | 361 | 8783** | 13.1 | 4253** | 16.0 | 0.58 |
|  | **ICMP 91751** | 180 | 300 | 392 | 7276** | 11.9 | 0.80 |  | 190 | 262 | 397 | 4866** | 9.8 | 0.82 |  | 231 | 285 | 341 | 8136** | 12.6 | 3916** | 15.4 | 0.60 |
|  | **ICMV 93752** | 218 | 291 | 394 | 4823** | 9.7 | 0.74 |  | 166 | 245 | 335 | 3738** | 8.6 | 0.75 |  | 229 | 274 | 334 | 5140** | 10.0 | 3407** | 12.9 | 0.51 |
|  | **UCC 23** | 238 | 346 | 457 | 7183** | 11.9 | 0.80 |  | 199 | 278 | 359 | 3762** | 8.6 | 0.69 |  | 259 | 308 | 373 | 6609** | 11.4 | 4233** | 14.6 | 0.52 |
|  | **CO (Cu) 9** | 176 | 344 | 426 | 8362** | 12.8 | 0.80 |  | 207 | 276 | 375 | 4339** | 9.2 | 0.69 |  | 224 | 306 | 375 | 11095** | 14.7 | 3354** | 16.8 | 0.67 |
|  | **Overall** | 176 | 322 | 488 | 8862** | 13.2 | 0.82 |  | 166 | 268 | 397 | 4327** | 9.2 | 0.75 |  | 223 | 295 | 375 | 9377** | 13.6 | 4046** | 16.2 | 0.62 |

| **Trait** | **Population** | **Rainy season 2005** | | | | | |  | **Summer season 2006** | | | | | |  | **Across-seasons** | | | | | | | |
| --- | --- | --- | --- | --- | --- | --- | --- | --- | --- | --- | --- | --- | --- | --- | --- | --- | --- | --- | --- | --- | --- | --- | --- |
|  |  | **Min** | **Mean** | **Max** | **σ^2^g** | **SE**  **(σ^2^g)** | ***h^2^*** |  | **Min** | **Mean** | **Max** | **σ^2^g** | **SE**  **(σ^2^g)** | ***h^2^*** |  | **Min** | **Mean** | **Max** | **σ^2^g** | **SE**  **(σ^2^g)** | **σ^2^gs** | **SE**  **(σ^2^gs)** | ***h^2^*** |
| **BMY** | **ICMP 87750** | 413 | 594 | 817 | 23617** | 21.5 | 0.75 |  | 385 | 504 | 650 | 9808** | 13.9 | 0.69 |  | 460 | 555 | 654 | 22285** | 20.9 | 11301** | 25.7 | 0.56 |
|  | **ICMP 91751** | 423 | 586 | 723 | 16462** | 18.0 | 0.72 |  | 346 | 507 | 687 | 11263** | 14.9 | 0.74 |  | 479 | 554 | 628 | 16851** | 18.2 | 10734** | 23.3 | 0.52 |
|  | **ICMV 93752** | 455 | 583 | 719 | 12814** | 15.9 | 0.72 |  | 382 | 501 | 667 | 9442** | 13.6 | 0.74 |  | 486 | 550 | 621 | 12710** | 15.8 | 9435** | 20.8 | 0.48 |
|  | **UCC 23** | 468 | 651 | 815 | 16547** | 18.0 | 0.75 |  | 328 | 513 | 647 | 11121** | 14.8 | 0.68 |  | 495 | 578 | 643 | 15554** | 17.5 | 12159** | 23.3 | 0.47 |
|  | **CO (Cu) 9** | 464 | 657 | 786 | 18478** | 19.0 | 0.70 |  | 379 | 516 | 673 | 12623** | 15.7 | 0.72 |  | 501 | 581 | 666 | 21517** | 20.5 | 9572* | 24.7 | 0.57 |
|  | **Overall** | 413 | 619 | 817 | 20540** | 20.1 | 0.76 |  | 328 | 511 | 687 | 10455** | 14.3 | 0.71 |  | 460 | 566 | 666 | 19405** | 19.5 | 11532** | 24.6 | 0.53 |
|  |  |  |  |  |  |  |  |  |  |  |  |  |  |  |  |  |  |  |  |  |  |  |  |
| **TGM** | **ICMP 87750** | 8.03 | 9.58 | 11.30 | 2.47** | 0.22 | 0.82 |  | 7.15 | 8.55 | 10.50 | 1.59** | 0.18 | 0.74 |  | 7.98 | 9.05 | 10.48 | 3.16** | 0.25 | 0.90** | 0.28 | 0.68 |
|  | **ICMP 91751** | 8.04 | 9.55 | 11.24 | 2.14** | 0.20 | 0.81 |  | 6.22 | 8.23 | 9.77 | 1.86** | 0.19 | 0.83 |  | 7.75 | 8.91 | 10.06 | 3.06** | 0.25 | 0.93** | 0.28 | 0.68 |
|  | **ICMV 93752** | 7.39 | 9.47 | 12.12 | 3.46** | 0.26 | 0.87 |  | 6.65 | 8.39 | 10.15 | 1.58** | 0.18 | 0.75 |  | 7.55 | 8.94 | 10.52 | 4.04** | 0.28 | 1.01** | 0.31 | 0.72 |
|  | **UCC 23** | 7.89 | 9.29 | 11.22 | 2.33** | 0.21 | 0.80 |  | 6.81 | 8.29 | 9.87 | 1.29** | 0.16 | 0.69 |  | 8.01 | 8.83 | 10.27 | 2.70** | 0.23 | 0.92* | 0.27 | 0.63 |
|  | **CO (Cu) 9** | 7.05 | 9.62 | 11.93 | 4.31** | 0.29 | 0.87 |  | 6.63 | 8.30 | 10.35 | 2.04** | 0.20 | 0.77 |  | 7.59 | 8.97 | 10.36 | 4.60** | 0.30 | 1.76** | 0.35 | 0.65 |
|  | **Overall** | 7.05 | 9.53 | 12.12 | 2.85** | 0.24 | 0.83 |  | 6.22 | 8.38 | 10.50 | 1.64** | 0.18 | 0.75 |  | 7.55 | 8.96 | 10.52 | 3.40** | 0.26 | 1.09** | 0.30 | 0.67 |
|  |  |  |  |  |  |  |  |  |  |  |  |  |  |  |  |  |  |  |  |  |  |  |  |
| **PGN** | **ICMP 87750** | 1347 | 2004 | 2701 | 307526** | 77.65 | 0.74 |  | 1434 | 2036 | 3117 | 319912** | 79.20 | 0.73 |  | 1609 | 2033 | 2613 | 434981** | 92.35 | 190168** | 110.72 | 0.59 |
|  | **ICMP 91751** | 1117 | 1831 | 2638 | 427198** | 91.52 | 0.87 |  | 1229 | 2026 | 2813 | 322094** | 79.47 | 0.74 |  | 1330 | 1955 | 2469 | 565234** | 105.28 | 186055** | 121.37 | 0.67 |
|  | **ICMV 93752** | 1261 | 1891 | 2581 | 313402** | 78.39 | 0.81 |  | 1244 | 1978 | 3004 | 310895** | 78.08 | 0.74 |  | 1598 | 1966 | 2583 | 466589** | 95.65 | 153976** | 110.31 | 0.66 |
|  | **UCC 23** | 1554 | 2253 | 3205 | 521128** | 101.09 | 0.83 |  | 1583 | 2233 | 2777 | 277796** | 73.80 | 0.64 |  | 1763 | 2218 | 2789 | 609310** | 109.30 | 192896* | 125.42 | 0.65 |
|  | **CO (Cu) 9** | 1412 | 2194 | 3042 | 542883** | 103.17 | 0.86 |  | 1487 | 2195 | 3287 | 352503** | 83.14 | 0.72 |  | 1724 | 2176 | 2823 | 646470** | 112.59 | 246434** | 132.32 | 0.64 |
|  | **Overall** | 1117 | 2054 | 3205 | 487189** | 97.74 | 0.85 |  | 1229 | 2107 | 3287 | 338002** | 81.41 | 0.73 |  | 1330 | 2081 | 2823 | 627865** | 110.96 | 196027** | 127.10 | 0.67 |
|  |  |  |  |  |  |  |  |  |  |  |  |  |  |  |  |  |  |  |  |  |  |  |  |
| **VGI** | **ICMP 87750** | 6.81 | 9.23 | 11.96 | 5.00** | 0.31 | 0.72 |  | 5.66 | 7.30 | 9.68 | 2.08** | 0.20 | 0.66 |  | 7.31 | 8.40 | 9.51 | 4.58** | 0.30 | 2.56** | 0.37 | 0.53 |
|  | **ICMP 91751** | 7.16 | 9.49 | 11.73 | 3.64** | 0.27 | 0.66 |  | 5.04 | 7.44 | 9.62 | 2.33** | 0.21 | 0.72 |  | 7.53 | 8.53 | 9.60 | 3.74** | 0.27 | 2.20** | 0.34 | 0.51 |
|  | **ICMV 93752** | 7.51 | 9.40 | 11.42 | 2.63** | 0.23 | 0.65 |  | 5.77 | 7.46 | 9.97 | 2.12** | 0.20 | 0.72 |  | 7.71 | 8.49 | 9.25 | 2.48** | 0.22 | 2.25** | 0.30 | 0.42 |
|  | **UCC 23** | 6.97 | 9.92 | 12.21 | 3.30** | 0.25 | 0.69 |  | 4.90 | 7.38 | 9.19 | 2.48** | 0.22 | 0.69 |  | 7.45 | 8.62 | 9.41 | 3.52** | 0.26 | 2.22** | 0.34 | 0.50 |
|  | **CO (Cu) 9** | 6.85 | 10.06 | 11.75 | 3.50** | 0.26 | 0.64 |  | 5.50 | 7.43 | 9.76 | 2.49** | 0.22 | 0.71 |  | 7.52 | 8.68 | 9.57 | 3.90** | 0.28 | 2.09^ns^ | 0.34 | 0.52 |
|  | **Overall** | 6.81 | 9.69 | 12.21 | 3.88** | 0.28 | 0.69 |  | 4.90 | 7.44 | 9.97 | 2.21** | 0.21 | 0.69 |  | 7.31 | 8.57 | 9.60 | 3.71** | 0.27 | 2.37** | 0.35 | 0.50 |
|  |  |  |  |  |  |  |  |  |  |  |  |  |  |  |  |  |  |  |  |  |  |  |  |
| **HI** | **ICMP 87750** | 27.5 | 34.8 | 40.0 | 21.20** | 0.64 | 0.74 |  | 19.9 | 31.5 | 40.5 | 40.77** | 0.89 | 0.80 |  | 30.2 | 33.4 | 36.7 | 34.80** | 0.83 | 27.31** | 1.10 | 0.49 |
|  | **ICMP 91751** | 28.4 | 35.4 | 41.0 | 26.08** | 0.72 | 0.80 |  | 27.4 | 32.7 | 39.3 | 27.46** | 0.73 | 0.64 |  | 30.1 | 34.0 | 38.0 | 32.82** | 0.80 | 20.81** | 1.03 | 0.51 |
|  | **ICMV 93752** | 30.8 | 36.7 | 40.6 | 14.32** | 0.53 | 0.67 |  | 25.2 | 34.6 | 41.1 | 22.76^ns^ | 0.67 | 0.58 |  | 32.4 | 35.1 | 37.4 | 19.69** | 0.62 | 17.97* | 0.86 | 0.40 |
|  | **UCC 23** | 30.0 | 34.5 | 39.0 | 11.62** | 0.48 | 0.66 |  | 24.0 | 31.2 | 37.7 | 29.86** | 0.77 | 0.64 |  | 29.5 | 33.2 | 36.7 | 29.02** | 0.75 | 12.38^ns^ | 0.90 | 0.55 |
|  | **CO (Cu) 9** | 30.6 | 34.8 | 38.4 | 10.37** | 0.45 | 0.66 |  | 24.1 | 31.5 | 37.5 | 19.68* | 0.62 | 0.62 |  | 31.0 | 33.4 | 36.1 | 17.67** | 0.59 | 12.48* | 0.77 | 0.46 |
|  | **Overall** | 27.5 | 35.3 | 41.0 | 17.95** | 0.59 | 0.74 |  | 19.9 | 32.3 | 41.1 | 32.51** | 0.80 | 0.70 |  | 29.5 | 33.8 | 38.0 | 32.61** | 0.80 | 17.93** | 1.00 | 0.54 |

*Significance at 0.05 level of probability, **Significance at 0.01 level of probability, ^ns^non-significant;

FT - Flowering time (days), PH - Plant height (cm), PL - Panicle length (cm), PD - Panicle diameter (mm), HC - Head count per m^2^, ET - Effective tillers per plant, PY - Panicle yield (g/m^2^), GY - Grain yield (g/m^2^), PTP - Panicle threshing percentage (%), SDMY - Stover dry matter yield (g/m^2^), BMY - Biomass yield (g/m^2^), TGM - 1000-grain mass (g), PGN - Panicle grain number, VGI – Vegetative growth index (g/m^2^ /day), HI - Harvest index (%)


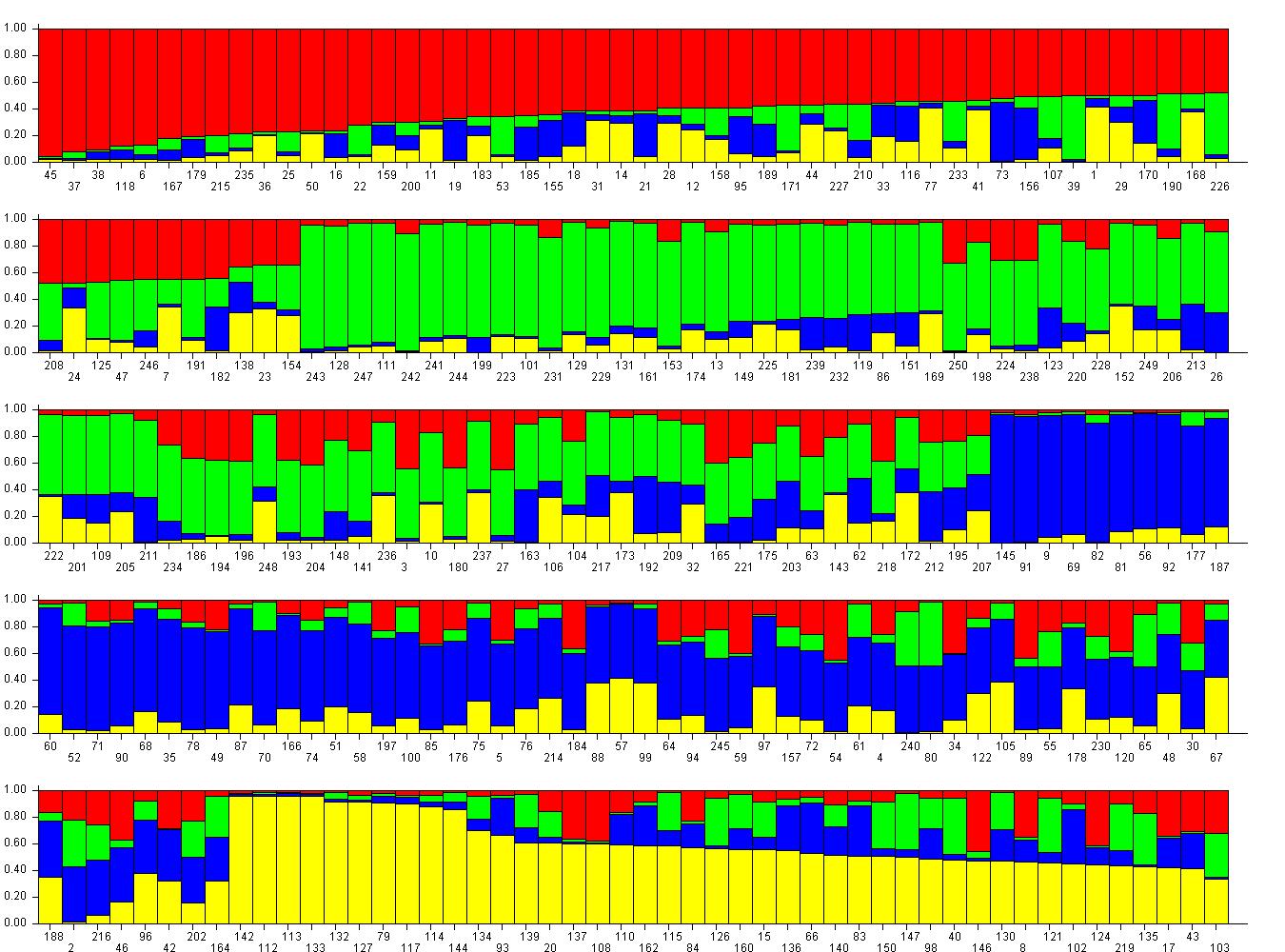


**G=1**

**G=2**

**G=3**

**G=4**

Supplementary file 4: Estimated population structure of 250 FS lines (*K* = 4). The y-axis is the group (sub-population) membership, and the x-axis is the individual FS genotypes in each of the five sampled pearl millet populations [1-50 from ICMP 87750; 51-100 from ICMP 91751; 101-150 from ICMV 93752; 151-200 from UCC 23, and 201-250 from CO (Cu) 9]. G (G1–G4) stands for a group = sub-population.

Supplementary file 5a: Association of SSR marker alleles with phenotypic traits using GLM and MLM (Q+K) models in the rainy season 2005 BLUPs.

| Trait | Locus | Linkage group | Correlation with phenotype | GLM Model | | MLM (Q+K) Model | |
| --- | --- | --- | --- | --- | --- | --- | --- |
|  |  |  |  | F ratio | -log10  p value | F ratio | -log10  p value |
| FT | *Xicmp*3027_202 | 5 | 0.31 | 4.15 | 2.91 | 1.62 | ns |
|  | *Xpsmp*2077_136 | 2 | -0.02 | 4.12 | 2.52 | 1.35 | ns |
|  | *Xpsmp*2085_175 | 4 | 0.24 | 3.73 | 2.24 | 3.30 | ns |
|  | *Xpsmp*2088_136 | 2 | 0.00 | 3.67 | 2.50 | 2.17 | ns |
|  | *Xpsmp*2090_173 | 1 | 0.11 | 3.65 | 2.18 | 2.26 | ns |
|  | *Xpsmp*2220_115 | 5 | 0.00 | 3.34 | 2.21 | 1.92 | ns |
|  | *Xpsmp*2224_159 | 7 | -0.22 | 3.59 | 2.14 | 0.92 | ns |
|  | *Xpsmp*2227_194 | 3 | 0.20 | 3.51 | 2.36 | 2.35 | ns |
|  | *Xpsmp*2248_162 | 6 | -0.31 | 8.70 | 5.85 | 3.53 | 2.09 |
|  | *Xpsmp*2248_166 | 6 | 0.32 | 6.50 | 4.26 | 2.06 | ns |
| PH | *Xicmp*3027_202 | 5 | 0.36 | 4.95 | 3.61 | 1.75 | ns |
|  | *Xpsmp*2077_136 | 2 | -0.03 | 4.91 | 3.10 | 2.18 | ns |
|  | *Xpsmp*2085_175 | 4 | 0.31 | 6.22 | 4.06 | 4.61 | 2.88 |
|  | *Xpsmp*2220_111 | 5 | -0.21 | 3.57 | 2.41 | 2.24 | ns |
|  | *Xpsmp*2224_157 | 7 | 0.26 | 5.84 | 3.78 | 4.82 | 3.04 |
|  | *Xpsmp*2224_159 | 7 | -0.29 | 4.79 | 3.01 | 1.96 | ns |
|  | *Xpsmp*2233_256 | 5 | 0.21 | 4.78 | 3.46 | 1.97 | ns |
|  | *Xpsmp*2233_260 | 5 | -0.06 | 3.10 | 2.00 | 2.11 | ns |
|  | *Xpsmp*2233_262 | 5 | -0.22 | 5.07 | 3.22 | 2.04 | ns |
|  | *Xpsmp*2248_162 | 6 | -0.34 | 7.43 | 4.94 | 3.53 | 2.10 |
|  | *Xpsmp*2248_166 | 6 | 0.34 | 6.48 | 4.25 | 2.46 | ns |
| PL | *Xpsmp*2077_136 | 2 | 0.08 | 4.19 | 2.58 | 3.53 | 2.09 |
|  | *Xpsmp*2233_260 | 5 | -0.10 | 3.77 | 2.58 | 2.62 | ns |
| PD | *Xpsmp*2069_210 | 1 | -0.16 | 3.18 | 2.07 | 2.10 | ns |
|  | *Xpsmp*2201_364 | 2 | 0.11 | 4.14 | 2.54 | 3.57 | 2.13 |
|  | *Xpsmp*2275_273 | 6 | 0.25 | 3.24 | 2.13 | 2.42 | ns |
| GY | *Xpsmp*2224_159 | 7 | -0.27 | 4.84 | 3.05 | 3.47 | 2.05 |
|  | *Xpsmp*2237_230 | 2 | 0.05 | 3.71 | 2.53 | 2.90 | ns |
| SDMY | *Xicmp*3027_202 | 5 | 0.25 | 3.19 | 2.08 | 1.88 | ns |
|  | *Xpsmp*2077_136 | 2 | -0.06 | 3.83 | 2.31 | 2.65 | ns |
|  | *Xpsmp*2085_175 | 4 | 0.25 | 4.10 | 2.51 | 2.49 | ns |
|  | *Xpsmp*2220_115 | 5 | 0.02 | 3.33 | 2.21 | 1.68 | ns |
|  | *Xpsmp*2224_157 | 7 | 0.20 | 3.41 | 2.01 | 3.19 | ns |
|  | *Xpsmp*2233_256 | 5 | 0.14 | 4.22 | 2.97 | 2.86 | ns |
|  | *Xpsmp*2233_262 | 5 | -0.11 | 3.96 | 2.41 | 3.11 | ns |
|  | *Xpsmp*2248_162 | 6 | -0.28 | 4.47 | 2.78 | 3.66 | 2.19 |
|  | *Xpsmp*2248_166 | 6 | 0.22 | 3.67 | 2.20 | 2.74 | ns |

Supplementary file 5a: Association of SSR marker alleles with phenotypic traits using GLM and MLM (Q+K) models in the rainy season 2005 BLUPs (Contd…).

| Trait | Locus | Linkage group | Correlation with phenotype | GLM Model | | MLM (Q+K) Model | |
| --- | --- | --- | --- | --- | --- | --- | --- |
|  |  |  |  | F ratio | -log10  p value | F ratio | -log10  p value |
| BMY | *Xpsmp*2077_136 | 2 | -0.05 | 3.87 | 2.34 | 2.61 | ns |
|  | *Xpsmp*2085_175 | 4 | 0.25 | 3.70 | 2.22 | 2.38 | ns |
|  | *Xpsmp*2220_115 | 5 | 0.05 | 3.17 | 2.07 | 1.82 | ns |
|  | *Xpsmp*2224_159 | 7 | -0.22 | 5.54 | 3.56 | 3.99 | 2.43 |
|  | *Xpsmp*2233_256 | 5 | 0.13 | 3.98 | 2.76 | 2.72 | ns |
|  | *Xpsmp*2233_262 | 5 | -0.15 | 4.03 | 2.46 | 2.91 | ns |
| PTP | *Xpsmp*2224_159 | 7 | -0.19 | 3.90 | 2.37 | 3.46 | 2.04 |
|  | *Xpsmp*2227_194 | 3 | 0.12 | 3.98 | 2.77 | 3.52 | 2.37 |
|  | *Xpsmp*2227_196 | 3 | -0.15 | 5.55 | 4.13 | 4.57 | 3.28 |
| TGM | *Xpsmp*2030_112 | 1 | 0.04 | 3.35 | 2.22 | 2.44 | ns |
| HI | *Xicmp*3027_200 | 5 | 0.26 | 3.59 | 2.42 | 1.65 | ns |
|  | *Xicmp*3027_202 | 5 | -0.24 | 3.17 | 2.07 | 1.34 | ns |
|  | *Xpsmp*2008_188 | 4 | -0.21 | 4.19 | 2.95 | 2.50 | ns |
|  | *Xpsmp*2027_229 | 7 | 0.27 | 3.72 | 2.54 | 3.34 | 2.21 |
|  | *Xpsmp*2027_233 | 7 | 0.23 | 3.78 | 2.27 | 2.10 | ns |
|  | *Xpsmp*2027_237 | 7 | -0.14 | 3.26 | 2.14 | 2.64 | ns |
|  | *Xpsmp*2224_159 | 7 | -0.11 | 3.02 | ns | 4.11 | 2.51 |
|  | *Xpsmp*2246_261 | 1 | 0.12 | 2.16 | ns | 3.20 | 2.09 |
|  | *Xpsmp*2248_162 | 6 | 0.29 | 3.64 | 2.17 | 1.87 | ns |
|  | *Xpsmp*2248_166 | 6 | -0.28 | 5.18 | 3.30 | 3.22 | ns |

FT - Flowering time, PH - Plant height, PL - Panicle length, PD - Panicle diameter, GY - Grain yield, SDMY - Stover dry matter yield, BMY - Biomass yield, PTP - Panicle threshing percentage, TGM - 1000-grain mass, HI - Harvest index

Supplementary file 5b: Association of marker alleles with phenotypic traits using GLM and MLM (Q+K) models in the summer season 2006 BLUPs.

| Trait | Locus | Linkage group | Correlation with phenotype | GLM Model | | MLM (Q+K) Model | |
| --- | --- | --- | --- | --- | --- | --- | --- |
|  |  |  |  | F ratio | -log10  p value | F ratio | -log10  p value |
| FT | *Xicmp*3027_202 | 5 | 0.24 | 3.52 | 2.37 | 1.69 | ns |
|  | *Xpsmp*2077_136 | 2 | 0.01 | 3.69 | 2.21 | 2.05 | ns |
|  | *Xpsmp*2088_136 | 2 | -0.07 | 3.59 | 2.42 | 2.22 | ns |
|  | *Xpsmp*2090_177 | 1 | 0.10 | 3.71 | 2.53 | 2.77 | ns |
|  | *Xpsmp*2248_162 | 6 | -0.21 | 4.66 | 2.92 | 2.46 | ns |
|  | *Xpsmp*2248_166 | 6 | 0.24 | 3.90 | 2.36 | 2.03 | ns |
| PH | *Xpsmp*2224_157 | 7 | 0.15 | 5.66 | 3.65 | 4.31 | 2.66 |
|  | *Xpsmp*2248_166 | 6 | 0.28 | 4.17 | 2.56 | 2.19 | ns |
| PL | *Xpsmp*2233_260 | 5 | -0.06 | 3.32 | 2.19 | 2.15 | ns |
| PD | *Xpsmp*2220_115 | 5 | 0.03 | 3.54 | 2.38 | 2.56 | ns |
| SDMY | *Xicmp*3058_193 | 6 | 0.04 | 4.05 | 2.82 | 3.22 | 2.11 |
|  | *Xpsmp*2076_160 | 4 | 0.22 | 3.23 | 2.11 | 2.01 | ns |
|  | *Xpsmp*2208_252 | 5 | 0.15 | 3.63 | 2.17 | 2.91 | ns |
|  | *Xpsmp*2224_157 | 7 | 0.13 | 3.78 | 2.28 | 2.70 | ns |
| BMY | *Xicmp*3058_193 | 6 | 0.04 | 3.17 | 2.07 | 1.35 | ns |
| PTP | *Xpsmp*2085_175 | 4 | 0.06 | 3.25 | ns | 3.41 | 2.01 |
|  | *Xpsmp*2201_364 | 2 | -0.11 | 3.31 | ns | 3.98 | 2.42 |
| TGM | *Xpsmp*2208_246 | 5 | -0.17 | 3.26 | 2.14 | 2.79 | ns |
|  | *Xpsmp*2277_242 | 5 | -0.06 | 3.39 | 2.26 | 3.52 | 2.36 |
| HI | *Xpsmp*2248_162 | 6 | 0.18 | 3.58 | 2.13 | 3.19 | ns |
|  | *Xpsmp*2248_166 | 6 | -0.20 | 4.01 | 2.44 | 3.54 | 2.10 |

FT - Flowering time, PH - Plant height, PL - Panicle length, PD - Panicle diameter, SDMY - Stover dry matter yield, BMY - Biomass yield, PTP - Panicle threshing percentage, TGM - 1000-grain mass, HI - Harvest index
